# Integrative Analysis of Three RNA Sequencing Methods Identifies Mutually Exclusive Exons of MADS-Box Isoforms During Early Bud Development in *Picea abies*

**DOI:** 10.3389/fpls.2018.01625

**Published:** 2018-11-13

**Authors:** Shirin Akhter, Warren W. Kretzschmar, Veronika Nordal, Nicolas Delhomme, Nathaniel R. Street, Ove Nilsson, Olof Emanuelsson, Jens F. Sundström

**Affiliations:** ^1^Linnean Center for Plant Biology, Uppsala BioCenter, Department of Plant Biology, Swedish University of Agricultural Sciences, Uppsala, Sweden; ^2^Science for Life Laboratory, Department of Gene Technology, School of Engineering Sciences in Biotechnology, Chemistry and Health, KTH Royal Institute of Technology, Solna, Sweden; ^3^Umeå Plant Science Centre, Department of Forest Genetics and Plant Physiology, Swedish University of Agricultural Sciences, Umeå, Sweden; ^4^Umeå Plant Science Centre, Department of Plant Physiology, Umeå University, Umeå, Sweden

**Keywords:** *Picea abies*, MADS-box genes, cone development, De Bruijn assembly, transcript isoforms, RNA sequencing, *DAL19*

## Abstract

Recent efforts to sequence the genomes and transcriptomes of several gymnosperm species have revealed an increased complexity in certain gene families in gymnosperms as compared to angiosperms. One example of this is the gymnosperm sister clade to angiosperm TM3-like MADS-box genes, which at least in the conifer lineage has expanded in number of genes. We have previously identified a member of this sub-clade, the conifer gene *DEFICIENS AGAMOUS LIKE 19 (DAL19)*, as being specifically upregulated in cone-setting shoots. Here, we show through Sanger sequencing of mRNA-derived cDNA and mapping to assembled conifer genomic sequences that *DAL19* produces six mature mRNA splice variants in *Picea abies.* These splice variants use alternate first and last exons, while their four central exons constitute a core region present in all six transcripts. Thus, they are likely to be transcript isoforms. Quantitative Real-Time PCR revealed that two mutually exclusive first *DAL19* exons are differentially expressed across meristems that will form either male or female cones, or vegetative shoots. Furthermore, mRNA *in situ* hybridization revealed that two mutually exclusive last DAL19 exons were expressed in a cell-specific pattern within bud meristems. Based on these findings in *DAL19*, we developed a sensitive approach to transcript isoform assembly from short-read sequencing of mRNA. We applied this method to 42 putative MADS-box core regions in *P. abies*, from which we assembled 1084 putative transcripts. We manually curated these transcripts to arrive at 933 assembled transcript isoforms of 38 putative MADS-box genes. 152 of these isoforms, which we assign to 28 putative MADS-box genes, were differentially expressed across eight female, male, and vegetative buds. We further provide evidence of the expression of 16 out of the 38 putative MADS-box genes by mapping PacBio Iso-Seq circular consensus reads derived from pooled sample sequencing to assembled transcripts. In summary, our analyses reveal the use of mutually exclusive exons of MADS-box gene isoforms during early bud development in *P. abies*, and we find that the large number of identified MADS-box transcripts in *P. abies* results not only from expansion of the gene family through gene duplication events but also from the generation of numerous splice variants.

## Introduction

In plants, members of the MADS-box gene family play important roles during diverse aspects of plant development and have been implicated in regulating e.g., floral transition and floral meristem and organ identity (see e.g., O’Maoileidigh et al., 2014 and references within). MADS is an acronym for the four founding members of this gene family: *MCM1* from *Saccharomyces cerevisiae, AGAMOUS* from *Arabidopsis thaliana, DEFICIENS* from *Antirrhinum majus*, and *SRF* from *Homo sapiens* (Schwarz-Sommer et al., 1990). MADS-box genes encode transcription factors. Evolutionary studies in seed plants have demonstrated that angiosperms and gymnosperms share orthologous MADS-box genes, and that these orthologs in many cases also are involved in similar biological processes. For a comprehensive review see Gramzow and Theissen (2010). For instance, MADS-box genes regulating carpel development in angiosperms have orthologous genes in gymnosperms involved in female cone development (Tandre et al., 1995; Winter et al., 1999).

We have previously produced inbred crosses of a naturally occurring spruce mutant *Picea abies* (var.) *acrocona* (Uddenberg et al., 2013). As reported, one quarter of the segregating sibling population resulting from those crosses initiated cones early, already during the second growth cycle, which suggests that genes of importance for vegetative to reproductive phase change are ectopically activated in the *acrocona* mutant. We have identified mature mRNA transcripts of the gene *DEFICIENS AGAMOUS LIKE 19 (DAL19)* as being specifically upregulated in cone-setting shoots of early cone-setting *acrocona* plants (Uddenberg et al., 2013). DAL19 belongs to the MIKC-type of MADS-box transcription factor proteins. MIKC refers to the different protein domains found in a class of plant-specific MADS-box genes (Gramzow and Theissen, 2010): M is short for the MADS-domain, which is responsible for DNA binding, I is a variable intervening region sometimes also referred to as the Linker, the K-domain is a keratin-like domain responsible for protein-protein interactions, and C is a variable C-terminal region (Ma et al., 1991), which in some MADS-box genes has been shown to encode activation domains (Litt and Irish, 2003).

Phylogenetic analyses have demonstrated that *DAL19*, together with closely related gymnosperm genes, form a distinct subclade in the MADS-box gene phylogeny (Uddenberg et al., 2013). The gymnosperm genes form a sister-clade to angiosperm *TM3*-like genes, which harbors the floral integrator *SUPPRESSOR OF OVEREXPRESSION OF CO 1 (SOC1)*, also known as *AGAMOUSLIKE20 (AGL20)* from *Arabidopsis thaliana* (Gramzow et al., 2014). SOC1 and its orthologs in other angiosperm species, integrate several flowering signals derived from e.g., temperature, aging, plant hormones, and photoperiod to regulate the transition from vegetative to reproductive phase (Dorca-Fornell et al., 2011), and (Gramzow et al., 2014) hypothesize that at least some members in the gymnosperm TM3 sister-clade perform similar functions.

The *DAL19* transcripts were first cloned by 3′ and 5′ Rapid Amplification of cDNA Ends (RACE), and by mRNA *in situ* hybridization shown to be preferentially expressed in the abaxial part of the ovuliferous scales in female cones (Carlsbecker et al., 2013). Sequence comparison between the *DAL19* clone (KC347015) presented in Carlsbecker et al. (2013) and the assembled *DAL19* transcript presented in Uddenberg et al. (2013) (*Acr42124_1*) revealed distinct sequence differences in the 5′ part of the transcript that are all contained within the first exon.

Mortazavi et al. (2008) showed that massively parallel short-read mRNA sequencing could be used together with a well-annotated reference genome to map reads across splice junctions to identify novel transcript isoforms in mouse and human. Later studies in plants employed reference-guided assembly of short-read mRNA sequencing to annotate reference genomes with new alternatively spliced transcript isoforms in *Arabidopsis thaliana* (Marquez et al., 2012), *Zea mays* (Thatcher et al., 2014), and *Brachypodium distachyon* (Mandadi and Scholthof, 2015). However, in organisms where high-quality reference genomes are not available, *de novo* transcriptome assembly (Robertson et al., 2010; Grabherr et al., 2011; Schulz et al., 2012) is the only approach available for transcript isoform reconstruction from short reads (Conesa et al., 2016). Unfortunately, current methods for *de novo* transcriptome assembly have difficulties reconstructing full-length transcript isoforms (Steijger et al., 2013; Xu et al., 2015; Conesa et al., 2016). Several recent plant studies adopted a hybrid sequencing approach where long, error-corrected reads derived from Pacific Biosciences’ isoform sequencing technology, Iso-Seq, were used to identify novel transcripts, and short, high-quality reads from massively parallel mRNA sequencing were used to correct and quantify those transcripts by reference-guided assembly (Xu et al., 2015; Abdel-Ghany et al., 2016; Wang et al., 2016; Liu et al., 2017) or by reference-free assembly (Hoang et al., 2017).

Hybrid sequencing studies of organisms with a well-annotated reference genome found evidence that the inclusion of Iso-Seq reads increased the sensitivity to alternatively splicing transcript isoforms. In root tissue of *Salvia miltiorrhiza*, Xu et al. (2015) reported detecting 110,715 and 1,109,011 known splice junctions with Illumina short reads and Iso-Seq, respectively. Using just Iso-Seq reads, Abdel-Ghany et al. (2016) increased the number of known transcript isoforms in sorghum from 2,950 to 10,053, and Wang et al. (2016) increased the number of known transcript isoforms in *Zea mays* from 63,540 to 111,151. However, when compared on a transcriptome coverage basis, long reads from Iso-Seq are substantially more expensive than Illumina short reads, and this is reflected in the lack of long-read sequencing depth to identify uncommon transcripts in both Liu et al. (2017) and Hoang et al. (2017). For example, Liu et al. (2017) found that their Iso-Seq data only covered 62% of the multi-exonic genes in their chosen reference gene model, and Hoang et al. (2017) found that only 87% of Illumina short reads mapped back to transcript isoforms derived from Iso-Seq.

In this study, we only had Iso-Seq reads from pooled sample sequencing available, but our interest was in exploring the transcript isoforms in a subset of those samples for which we had short reads from total mRNA sequencing (RNA-seq). Lacking a finished reference genome for *P. abies* we tried assembling our short reads with Trinity (Grabherr et al., 2011) and Oases (Schulz et al., 2012), two popular assemblers built around De Bruijn graphs. Unfortunately, these methods did not reconstruct *DAL19* isoforms well, so we developed a novel approach that combines naive assembly from a De Bruijn graph with kallisto (Bray et al., 2016). We use this approach to construct a parsimonious set of full-length transcript isoforms for *DAL19.*

We verify the presence of multiple mature mRNA transcript isoforms of *DAL19* using three independent methods: (i) Rapid Amplification of cDNA Ends (RACE) followed by Sanger sequencing (Yeku and Frohman, 2011), (ii) a novel method for *de novo* assembly of short RNA-seq reads *in silico*, and (iii) PacBio Iso-Seq RNA sequencing (Sharon et al., 2013). Furthermore, we map the *DAL19* transcripts derived from Sanger sequencing to the genome sequences of *P. abies* and the closely related *Picea glauca* to address whether these *DAL19* transcripts are transcribed from one single locus, and hence can be considered transcript isoforms, or if these transcripts are transcribed from multiple loci.

In *P. abies*, female and male cones and vegetative shoots are initiated as buds on the growing shoots, i.e., female and male cones are formed from separate meristems (Hannerz and Sundström, 2006). Differential expression of the different isoforms in these tissues would be evidence that these isoforms are biologically relevant. Therefore, to address if the *DAL19* variants play distinct roles during development, we assay their transcription in young developing buds using quantitative Real-Time PCR (qRT-PCR) and mRNA *in situ* hybridization methods.

Finally, we address whether the observed pattern of multiple splice variants of *DAL19* is also present in other members of the MADS-box gene family in *P. abies*. Using our novel method for *de novo* assembly of short RNA-seq reads *in silico* and using long-read PacBio Iso-Seq RNA-seq data, we provide evidence for extensive alternative splicing of mutually exclusive exons among *P. abies* MADS-box genes that form a sister clade to angiosperm *TM3*-like genes. These findings suggest that vegetative to reproductive phase change and cone setting in *P. abies* are regulated by a genetic mechanism fine-tuned by the usage of different MADS-box isoforms in different bud types.

## Materials and Methods

### Plant Material

Plant material was collected from an adult tree of Norway spruce (*Picea abies* L. Karst.) at the Rörby seed orchard (latitude 59°54′ N) outside of Uppsala, Sweden. Male, female, and vegetative buds were collected during two distinct developmental phases of early bud development: (i) meristematic buds before or at the onset of lateral organ formation, (ii) buds that had progressed into active lateral organ formation and cell differentiation. Meristematic buds were collected on August 1st 2016. Lateral organ initiating buds were collected on August 16th 2015 or on September 12th 2016. In our study, a biological replicate consisted of a pool of three buds of one bud type from one adult tree. All plant materials used for RNA preparations were snap-frozen in liquid nitrogen and stored at –70°C. Plant materials used for *in situ* hybridization experiments were directly collected into fixative media according to Karlgren et al. (2009).

Samples for PacBio Iso-Seq sequencing were collected from several tissue types and conditions to give a broad representation of transcripts expressed in *Picea abies*. In total, 33 samples were collected and the tissue types included: (i) developmental samples of roots, hypocotyl, SAM and cotyledons, vegetative-, male- and female buds, pollen, ovuliferous scales and ovules, (ii) diurnal samples of cambial tissue, (iii) vascular samples of xylem and phloem, and (iv) cold stress samples of roots. All samples collected for PacBio Iso-Seq sequencing were snap-frozen in liquid nitrogen and stored at –70°C. Total RNA was prepared as described below (see RNA Preparation) and the resulting RNA-samples were pooled before performing PacBio Iso-Seq sequencing (see RNA Sequencing). We describe the samples in Supplementary Table S1.

### RNA Preparation

Tissue homogenization, extraction, CHISAM (chloroform/isoamylalcohol 24:1) purification and isopropanol precipitation CHhomoRNA were performed as described by Azevedo et al. (2003). Resulting RNA pellets were dissolved in 350 μl RLT buffer (Qiagen RNeasy Kit) and long mRNAs were separated from microRNAs according to manufacturer’s instructions (Qiagen RNeasy Kit, Qiagen, Carlsbad, CA, United States).

### Cloning of *DAL19* Transcripts

Alternative mature mRNA transcript ends of *DAL19* were verified by synthesizing cDNA ends from mixed bud tissues (male, female and vegetative) and performing 5′ RACE and 3′ RACE according to the manufacturer protocol (FirstChoice^®^ RLM-RACE Kit, Thermo Fisher Scientific, Vilnius, Lithuania). Primers that were used in this approach are listed in Supplementary Tables S2B,C.

Isolated alternative cDNA ends from 5′ RACE and 3′ RACE were cloned into the PCR blunt II Topo vector and transformed into *Escherichia coli* according to manufacturer instructions (Zero Blunt^®^ TOPO^®^ PCR Cloning Kit, Invitrogen, Carlsbad, CA, United States). Transformants that carried the correct insert were selected using colony-PCR with gene specific primers and RACE primers provided by the kit listed in Supplementary Table S2. Plasmids were prepared using the GeneZet plasmid mini prep kit according to manufacturer instruction (Thermo Fisher Scientific, Vilnius, Lithuania). All plasmids were sent to GATC Biotech, Germany, for sequence verification.

### Quantitative Real-Time PCR

qRT-PCR was performed using CFX-connect Real-Time System on 96-well PCR plates with adhesive seals (Bio-Rad Laboratories). Expression was analyzed in three distinct biological samples of each tissue type, except for vegetative buds collected on September 12th 2016 in which one sample was lost due to RNA degradation. Each biological sample was analyzed in triplicate. Primers used to quantify expression levels are provided in Supplementary Table S2D. The expression data of each gene was normalized against the expression of three reference genes, *ACTIN, POLYUBIQUITIN*, and *HISTONE2A.* PCR amplifications were carried out using the Maxima SYBR Green/ROX qPCR kit (ThermoScientific, Vilnius, Lithuania). Total 10 ng cDNA was used in 25 μl PCR reaction. PCR cycling conditions were followed by the manufacturer instructions. The reactions were run for 40 cycles. Melt curves were generated to ensure product uniformity at the end of each run. Inter-run connector samples were included in all studies to correct for the use of multiple plates. Calculations and normalizations were done using the CFX software based on the “ΔCt or ΔΔCt methods” (Bio-Rad).

Statistical analyses were performed using R 3.4.2. Normalized gene expression values from qRT-PCR experiments were used as an input dataset in the analyses. Student’s *t*-test was performed to assess the significance of differences of gene expression between samples.

### *In situ* Hybridization

Tissue fixation, embedding, sectioning, *in situ* section pre-treatment, and preparation of hybridization solutions were performed as described by Karlgren et al. (2009). LNA probes with complementary sequences to *DAL19* variants were synthesized, 5′ and 3′ digoxigenin-labeled (Exiqon) (Supplementary Table S2E), and then hybridized at 52°C. *In situ* post hybridization was performed as described previously (Karlgren et al., 2009). The images of hybridization signals were taken using Zeiss Axioplan microscope equipped with an AxioCam ICc 5 camera.

### RNA Sequencing

2 × 125 bp short-read paired-end RNA sequencing of mRNA-enriched male, female, and vegetative bud samples collected on August 1st 2016 was performed using a HiSeq2500 with v4-sequencing chemistry by The SNP & SEQ Technology Platform in Uppsala, Sweden. We pre-processed all reads by removing ribosomal RNA with SortMeRNA version 2.1b (Kopylova et al., 2012), and by removing adapters and low-quality parts of reads with Trimmomatic version 0.36 (Bolger et al., 2014). The complete command can be found in Supplementary File S1.

The SNP & SEQ Technology Platform in Uppsala, Sweden, performed PacBio Iso-Seq sequencing of the pooled samples on a PacBio Sequel System. They processed the raw reads with the SMRTLink v5.0.1 Iso-Seq workflow to obtain circular consensus (CCS) reads. We present the subset of reads associated with *DAL1*-*DAL41*. Remaining reads will be made available at the conifer genomic resource database^1^.

### Discovery of Putative MADS-Box Core Region Sequences

We compiled a list of 32 previously published *P. abies* MADS-box genes (Carlsbecker et al., 2013) as well as ten newly identified full-length MIKC MADS-box genes retrieved from PlantGenIE.org (Sundell et al., 2015), here denoted *DAL31*- *DAL41* (Supplementary Table S3). We identified the full length MIKC MADS-box genes by performing BLAST searches using the option BLASTN- nucleotide query to nucleotide db and selecting blast DB *P. abies* 1.0 (complete) (Sundell et al., 2015). BLAST searches resulted in several scaffolds aligning to each cDNA sequence. We identified conserved intron/exon borders in the cDNA sequences by comparing the exon organization of spruce MADS-box genes to each other. The position of the first conserved intron/exon border is situated in the 3′ region of the MADS-domain. Similarly, we found a conserved intron/exon border that exists at the end of the K-domain of each MADS-box gene of *P. abies*. We defined core region sequences of *P. abies* MADS-box genes as starting at the first conserved intron/exon border and ending at the last conserved intron/exon border of the K-domain (Supplementary File S2).

### Assembly, Evaluation, and Visualization of *DAL19* Transcripts

We tested and developed RNA-seq assembly methods on a single training sample of vegetative *P. abies* buds. We created the Trinity (Grabherr et al., 2011) assembly from all reads that had a mate after trimming with Trimmomatic using Trinity version 2.4.0 (see Supplementary Listing S1). We created an Oases (Schulz et al., 2012) assembly from trimmed reads that had been merged with Pear version 0.9.10 (Zhang et al., 2014) using the “--keep-original” flag, and using Velvet 1.2.10 with Oases version 0.2.09.

We created an assembly using our own method: With Mccortex version v0.0.3-554-ga7d6f3b (Turner et al., 2018), we built and inferred a colored De Bruijn graph of *k*-mer size 47 from all reads that had a mate after trimming with Trimmomatic. We collapsed maximal runs of adjacent nodes with one incoming and one outgoing edge, excepting nodes at the ends of the runs, into unitigs. We removed unitigs with a mean coverage below four from the graph. We then traversed the resulting Cortex graph from every *k*-mer in *DAL19*_*ψ*, which resulted in one subgraph. We removed tips shorter than 47 *k*-mers from this subgraph. We created candidate transcripts from this cleaned graph by traversing every simple path from every incoming tip to every outgoing tip. Simple paths cannot contain cycles and therefore our candidate transcripts do not contain cycles either. For every sample, we assessed read support for all candidate transcripts with kallisto version 0.43.1 (Bray et al., 2016) from all reads that had a mate after trimming. We ran kallisto with 100 bootstrap samples. We kept for further analysis all candidate transcripts for which the number of estimated counts was one or more in at least 95 bootstrap samples.

**FIGURE 1:**
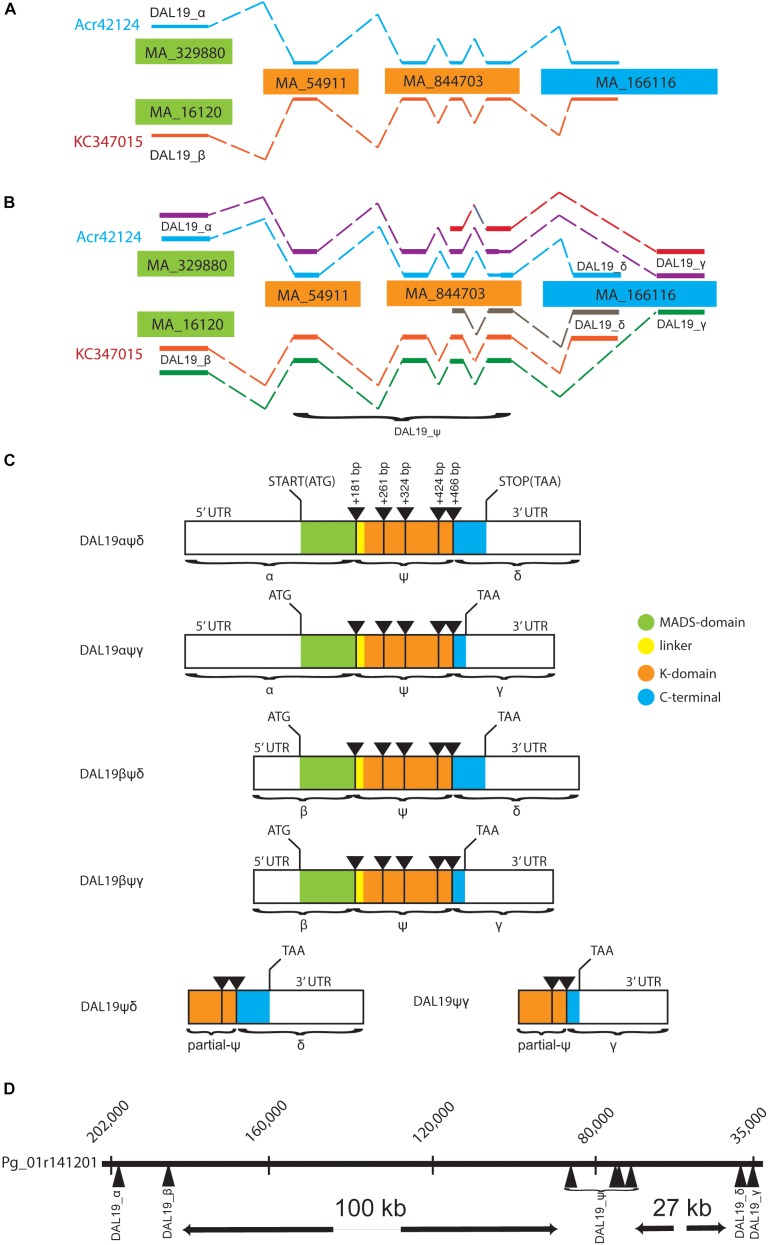
Schematic view of *DAL19* isoforms and their genomic organization. **(A)** The two transcripts Acr42124 and KC347015 mapped to *Picea abies* (V1) genome scaffolds. **(B)** The additional transcripts presented in the current manuscript are included. The *DAL19 exons* *α, β, δ*, and *γ*, and the core region *ψ* are indicated. **(C)** Illustration of the cloned *DAL19* isoforms. The MIKC-protein domain structure is color indicated, as well as the position of 5′ and 3′ UTRs, translational start and stop codons, and the position of intron/exon borders. **(D)** *DAL19* exons mapped to the *Picea glauca* genomic scaffold Pg01r141201.

We evaluated each transcriptome assembly method based on the length of the match of the transcript with the maximal match to each *DAL19* transcript isoform. We calculated the maximal match length for every assembly by mapping all reads to the four *DAL19* transcript isoforms αψγ, αψδ, βψγ, and βψδ with Minimap2 (default parameters) version 2.4-r555 (Li, 2018). See Figure 1C for a description of these isoforms. Match length was calculated for each transcript by counting the number of alignment matches in the alignment CIGAR string (SAMv1 specification^2^) and subtracting the number of base mismatches in the NM tag. A similarity proportion was calculated by dividing the length of the target *DAL19* transcript by the assembled transcript match length to that *DAL19* transcript.

We mapped all CCS reads obtained from the pooled sample to 42 core sequences of putative MADS-box genes in *P. abies* using Minimap2 version 2.4-r555 (Li, 2018) with the non-default argument “-x map-pb”. Twelve CCS reads had a primary alignment to one of the *DAL19* regions α, β, γ, δ, and ψ. We converted these reads to separate Cortex graphs of *k*-mer size 47. For each CCS-read graph, we joined the graph with the short-read graph and pruned unitigs with coverage below two. We then aggregated the twelve CCS reads by whether they had a primary or supplementary alignment to *DAL19_α, DAL19_β*, or neither of these two regions. Four CCS reads fell in each of those categories. We merged the four CCS-read graphs of each *DAL19*-region-based group into a single color. We joined the resulting three graphs with the short-read graph and five graphs consisting of the *k*-mers of the DAL19 regions α, β, γ, δ, and ψ.

**FIGURE 2:**
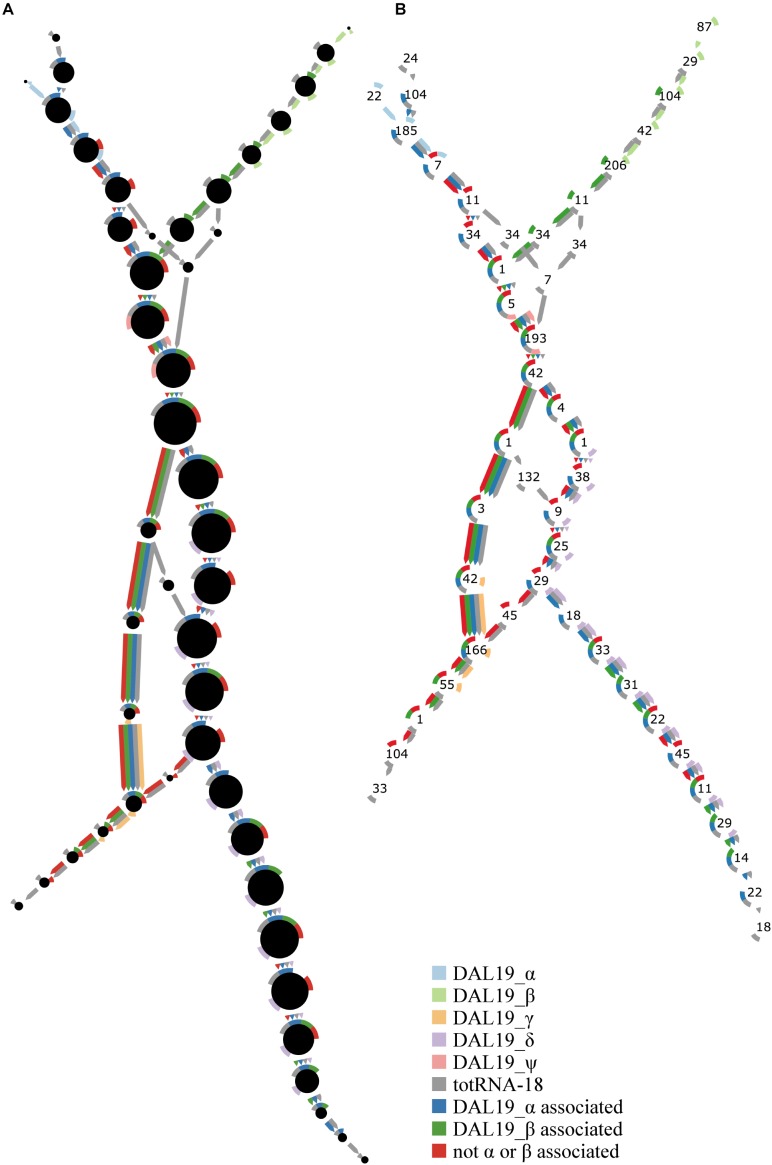
Complete traversal of final Cortex graph using all colors and starting from the first 47-mer of *DAL19_ψ*. **(A,B)** Represent the same graph topology. Colors represent different sources of sequencing data as defined in the figure. Circles represent unitigs. The rim of each circle consists of nine circular arcs of equal size representing one of each k-mer color. An arc is colored if the circle’s unitig has non-zero coverage of k-mers in that color. A colored edge connecting circles represents an edge between end k-mers of a unitig in the color of the edge. The first five light colors represent the *DAL19* exons α, β, γ, and δ, and the core region ψ (obtained from cloning followed by Sanger sequencing). Gray represents *k*-mers from short-read RNA-seq data of “totRNA18.” Dark blue represents four CCS reads that map to *DAL19_α*. Dark green represents four CCS reads that map to *DAL19_β*. Dark red represents four CCS reads that map to *DAL19_ψ*, but not the *α* or *β* region. **(A)** The area of the black central circle is proportional to the maximum of the per-color mean coverage of the unitig represented by that circle. **(B)** The number in the center of each circle is the number of k-mers that constitute the unitig represented by that circle.

For the purpose of visualization, the untig-filtered and tip-pruned Cortex graph was explored by traversing every connecting *k*-mer of any color from the initial 47-mer of *DAL19_ψ* using cortexpy version 0.23.5.^3^ The complete command can be found in Supplementary File S1. We visualized the graph traversal of the final Cortex graph (Figure 2) with the Javascript library D3 version 4 (Jasaitiene et al., 2011) and Circos (Krzywinski et al., 2009). We calculated the graph layout with a Javascript implementation of CoLa (Dwyer et al., 2006).

### Assembly and Filtering of MADS-Box Transcripts

For each of nine meristematic bud samples collected from a spruce tree on August 1st 2016 (three pools of male, vegetative, and female buds), we assembled transcript isoforms from pre-processed reads using our method (see Assembly, Evaluation, and Visualization of DAL19 Transcripts) with the difference that we traversed the unitig-filtered Cortex graph from every *k*-mer in the MADS-core sequences (see Discovery of Putative MADS-Box Core Region Sequences), which resulted in several subgraphs. For each subgraph, we removed tips shorter than 47 *k*-mers, created candidate transcripts, and filtered those transcripts with kallisto as described in Section “Assembly, Evaluation, and Visualization of DAL19 Transcripts.” We combined all transcripts assembled in this way across all nine samples in a single file while keeping a single copy of duplicate transcripts. Transcripts that are reverse complements of each other were considered to be duplicates for the purpose of this candidate transcript aggregation step. The resulting file contained 1084 sequences.

We annotated the combined set of assembled transcripts with putative protein domains using Translated Reverse Position Specific BLAST (“rpstblastn” from the “blast” package version 2.6.0, build Jan 2 2018) against the NCBI’s Conserved Domain Database (revised on 16 January 2015) (Marchler-Bauer et al., 2015) using an *e*-value threshold of 0.01. We kept for further analysis 1073 MADS-box transcript sequences that could be annotated with at least one protein domain.

### Assignment of Assembled MADS-Box Transcripts to MADS-Box Core Regions

We clustered the set of 1073 assembled transcripts that could be annotated with at least one protein domain using CD-HIT-EST version 4.6.4 (Li and Godzik, 2006) with the tuning parameters “-c 0.95 -n 10” in order to obtain clusters with sequence similarity above 95%. CD-HIT-EST provides a representative sequence for each cluster, and we mapped every cluster representative to our list of MADS-box core sequences using BWA-MEM version 0.7.8-r455 with default parameters. Finally, we assigned each assembled transcript in a cluster to the MADS-box core sequence that was the primary mapping of the cluster’s representative sequence. Thousand and forty assembled transcripts from 124 clusters could be assigned to 29 MADS-box core sequences in this way.

### Transcript Isoform Curation and Multiple Sequence Alignment

The 1040 assembled transcripts assigned to a specific MADS-box core region were translationally aligned using the ClustalW module within Geneious (Geneious pro version 10.2.3 created by Biomatters Ltd.)^4^. Using the alignment, transcript isoforms were identified by manual curation. BLASTN searches (Scoring matrix: BLOSUM62; *E*-value threshold: 1e-3) against the *P. abies* genome assembly V1.0^5^ were used to assign scaffolds from the genome assembly that contain 5′ or 3′ exons of MADS-box genes to each transcript isoform.

### Mapping of PacBio Circular Consensus Reads to MADS-Box Core Regions

Minimap2 version 2.4-r555 (Li, 2018) using the preset “map-pb” was used to map circular consensus (CCS) PacBio Iso-Seq reads to the assembled and filtered transcripts. Only alignments with an edit distance less than five were kept. The assignment of transcripts to MADS-box scaffolds described in Section “Assembly, Evaluation, and Visualization of DAL19 Transcripts.” was used to assign every CCS alignment to a MADS-box scaffold. Reads for which all primary and secondary alignments of a CCS read aggregated to the same MADS-box scaffold were counted in Table 1.

**Table 1:** Long read and differential expression analysis results aggregated by scaffold (MA ID).

*Gene name*	*MA_ID*	*Number of uniquely mapping CCS reads*	*Number of significant DE transcripts*	*Number of transcripts*
*DAL1*	MA_128164	20	15	80
*DAL1*	MA_369170	0	0	6
*DAL10*	MA_15122short	0	0	6
*DAL10*	MA_15122long	0	25	78
*DAL11*	MA_18048short	0	0	7
*DAL11*	MA_18048long	0	6	15
*DAL12*	MA_13933short	0	4	8
*DAL12*	MA_13933long	0	12	20
*DAL13*	MA_130755	7	9	25
*DAL13_like_a*	MA_2191711	0	2	4
*DAL13_like_b*	MA_8198323	0	0	3
*DAL19*	MA_16120	3	1	20
*DAL19*	MA_162822_long	0	13	47
*DAL19*	MA_329880	2	2	22
*DAL19*	MA_162822short	0	1	1
*DAL21*	MA_126898long	0	4	17
*DAL21*	MA_126898short	0	0	4
*DAL3*	MA_141872	10	6	23
*DAL3*	MA_10256834	0	6	32
*DAL3_like_a*	MA_19007	0	1	17
*DAL3_like_a*	MA_32676	1	1	16
*DAL31*	MA_46639	7	2	22
*DAL32*	MA_7858089	0	2	16
*DAL32*	MA_138440long	2	5	39
*DAL32*	MA_138440short	0	0	4
*DAL33*	MA_10079394	0	13	92
*DAL33*	MA_276701	3	1	31
*DAL35*	MA_9284799	1	3	20
*DAL37*	MA_18942	10	0	20
*DAL38*	A_20180long	6	1	11
*DAL38*	A_20180short	0	0	1
*DAL39*	MA_94049	0	4	25
*DAL4*	MA_101463	9	2	110
*DAL4*	MA_35712	0	1	29
*DAL40/JTL*	MA_10432602	3	0	14
*DAL41*	MA_57186	1	7	26
*DAL5*	MA_32019	0	0	3
*DAL9*	MA_6279308	5	3	19
*Sum*		90	152	933


*MA_ID: scaffold identification in *P. abies* genome assembly v1.0. Number of uniquely mapping CCS reads: Counts of long reads mapping uniquely to transcripts associated with a single scaffold. Number of significant DE transcripts: Number of significant associations at FDR 0.1 of transcript differential expression in eight bud samples, across bud types, and aggregated by MA ID. Of 38 groups of transcripts assigned to a single MA ID, 28 groups contained at least one transcript with differential expression across vegetative, male, and female buds at FDR 0.1. Number of transcripts: Number of transcripts assigned to each MA ID.*

### Estimation of Transcript Abundance With Kallisto

For each sample, we mapped read pairs processed only with SortMeRNA and Trimmomatic to the combined set of assembled transcripts using BWA-MEM version 0.7.8-r455 (Li, 2013) with default parameters and a minimum seed length of 47. We estimated the abundance of assembled transcripts with kallisto version 0.43.1 (default parameters) using only read pairs for which both reads mapped to any assembled transcript.

### Differential Expression Analysis

We converted estimated transcript counts calculated by kallisto to read counts using the tximport package (version 1.8.0) (Soneson et al., 2015). We compared samples based on all transcripts with a maximum read count greater than one (823 transcripts) by (i) calculating Spearman correlation coefficients of the raw transcript counts (Supplementary Figure S1), by (ii) creating a heatmap of log-scaled transcript read counts (Supplementary Figure S2), and by (iii) performing multi-dimensional scaling (Euclidean distance) (Supplementary Figure S3). Heatmaps were created using the function heatmap.2 from the gplots package (version 3.0.1, [warnes2016]) in R (version 3.5.0, [r_core_team]). In Supplementary Figures S1–S3 one male sample looks substantially different from the other two male samples. We removed this sample (“A43_S6_L001”) from the differential expression analysis of the assembled transcripts of nine bud samples.

We performed differential expression analysis on the converted read counts using a negative binomial Generalized Linear Model implemented in edgeR (version 3.22.1, R version 3.5.0) (Robinson et al., 2010) as a quasi-likelihood F-test (McCarthy et al., 2012) for any differences between all three vegetative bud groups. We used the Benjamini–Hochberg (Benjamini and Hochberg, 1995) procedure to correct *p*-values for false discovery rate. The results of differential expression analysis can be found in Supplementary Table S6.

### Phylogenetic Analyses

Two separate analyses were performed. In the first, the nucleotide sequence corresponding to the complete open reading frame (ORF) of representative transcript isoforms were translationally aligned using the ClustalW module within Geneious to annotated MADS-box genes retrieved from GenBank from the gymnosperms *Picea abies* and *Pinus radiata* and the angiosperms *Arabidopsis thaliana* and *Lycopersicum esculentum* (Supplementary Table S4 and Supplementary Files S3, S4). Phylogenetic parsimony analysis of the sequences (in total 116 taxa and 1385 characters) was performed using PAUP 4.0 a (build 161) (Swofford, 2002). A heuristic search with 1000 RANDOM additions was performed with the tree-bisection reconnection (TBR), steepest descent, and MULPARS options in effect. Support for the different groups in the tree was estimated using 100 bootstrap samples using the same heuristic search settings. In the second analysis, sequences outside of the MADS-box were trimmed away. The alignment of the MADS-box and subsequent parsimony analysis (120 taxa and 190 characters) was performed using the same settings as for the full length ORF.

## Results

### Cloning of *DAL19* Transcripts Reveals Four Novel Transcript Isoforms

In order to verify the presence of the different *DAL19* mature mRNA transcripts we first mapped the previously published *DAL19* transcripts, Acr42124 and KC347015, to the published *Picea abies* genome sequence (*Picea abies* v 1.0) (Nystedt et al., 2013). Due to the large genome size and prevalence of highly repetitive regions, scaffolds in the current genome assembly of *Picea abies* are relatively short and genes are commonly scattered over several genomic scaffolds. In line with this, both Acr42124 and KC347015 mapped to multiple genomic scaffolds (Figure 1A). Notably the sequence corresponding to the first exon of Acr42124 mapped to the genomic scaffold MA_329880 of 9.4 kbp, whereas the first exon of KC347015 mapped to the MA_16120 scaffold of 74 kbp (Figure 1B). We named these alternate exons *DAL19_α* and *DAL19_β*, respectively. The remaining exons mapped to the same set of genomic scaffolds: MA_54911, MA_844703, and MA_166116. In order to connect *DAL19_α* and *DAL19_β* with the adjacent exon on scaffold MA_54911, forward primers placed in the 5′ untranslated regions of either *DAL19_α* or *DAL19_β* were used in combination with reversed primers placed in the adjacent exon to amplify partial *DAL19* transcripts starting with either the *DAL19_α* or *DAL19_β* exons. Next, we used gene-specific *DAL19* primers in combination with generic 3′ poly-T or 5′CAP primers to amplify the 3′ or 5′ ends of *DAL19*. Surprisingly, this resulted in the amplification of a version of *DAL19* with an alternate 3′ end as compared to the previously published 3′ ends of KC347015 and Acr42124. The sequence that differs corresponds to the last exon of *DAL19* (Figure 1B). Both exons (henceforth called *DAL19_δ* and *DAL19_γ*, respectively) map to the same genomic scaffold (MA_166116) but at different positions. *DAL19_δ* maps to position 4650–4944 and harbors the C-terminal signature motif of the *TM3*-clade EVETQL, whereas *DAL19_γ* maps to position 3239–3505 and harbors a premature stop codon. In addition, the 5′ RACE yielded two short versions of *DAL19* that lacked the MADS-box altogether. In summary, six versions of *DAL19* have been cloned: four long versions with alternate first and last exons (*DAL19_αψδ, DAL19_αψγ, DAL19_βψδ*, and *DAL19_βψγ*), and two short versions (*DAL19_ψδ* and *DAL19_ψγ*), where *ψ* refers to a common core region with four exons, as detailed in Figure 1C. All versions have been independently cloned in their full-length versions and sequenced by Sanger sequencing.

### Mapping of *DAL19* Transcripts to Genomic Assemblies Provides Support That These Transcripts Are Isoforms

Given the fragmented assembly of the *P. abies* genome, it is not possible to rule out that we have identified *DAL19* transcripts from different genomic loci. However, the presence of both *DAL19_δ* and *DAL19_γ* on the same genomic scaffold, and the fact that *DAL19_α* and *DAL19_β* are expressed together with either *DAL19_δ* or *DAL19_γ*, supports the notion that the *DAL19* transcripts we observed are indeed transcribed from one large locus. To provide independent support for this hypothesis we mapped the cloned *DAL19* transcripts to the genomic assembly of *Picea glauca* (PG29-V4.0), with which *P. abies* shares sequence similarity both in terms of frequent synteny and sequence identity (Sundell et al., 2015). The *DAL19* transcripts all mapped to the same *Picea glauca* scaffold (Figure 1D). According to the *P. glauca* assembly, the two variants of the first exon of *PgDAL19* (*PgDAL19_α* and *PgDAL19_β*) are located approximately 16 kb apart and separated from the next *DAL19* exon by an intron of approximately 100 kb. The *DAL19_α* exon is located 5′ of the *DAL19_β* exon, which possibly has its own promoter inside the large first intron of *DAL19*. The introns between exons three, four, and five of *DAL19* are relatively short and consist of only approximately one hundred bases each. We consider this region the core region *ψ* of the *DAL19* gene (or *DAL19_ψ*) because the fifth exon is followed by a long intron of approximately 28 kb separating this region of *DAL19* from the two alternative 3′ exons *DAL19_δ* and *DAL19_γ*.

### Transcripts From Long-Read Sequencing and a Novel Transcriptome Assembly Method Are Consistent With Four DAL19 Transcript Isoforms

In order to provide independent evidence of all cloned DAL19 transcripts, we obtained short-read Illumina and long-read PacBio Iso-Seq circular consensus sequences (CCS) of total, long (>200 base pairs), polyadenylated RNA derived from a single sample of *P. abies* vegetative buds, and a pool of 33 *P. abies* samples, respectively. We represented the short reads as a De Bruijn graph, and then threaded the long reads through this graph. We then fully traversed the graph starting from the *DAL19* core region (*DAL19_ψ*). This traversal (Figure 2) shows short-read RNA-seq *k*-mer coverage for the *α, β, γ, δ*, and *ψ* regions of DAL19. Short-read coverage is split evenly between the *α* and *β* regions. When the *α* and *β* regions meet at *DAL19_ψ*, the short-read coverage merges additively. At the end of *DAL19_ψ*, the majority of short-read coverage follows *DAL19_δ*, and a minority of short-read coverage arrives at *DAL19_γ*. This uneven split of *k*-mer coverage between the *δ* and *γ* regions together with the even split of *k*-mer coverage between the *α* and *β* regions is inconsistent with the expression of less than three *DAL19* transcript isoforms.

We attempted to assemble these transcript isoforms from the short RNA-seq reads using Trinity and Oases. However, the proportion of known *DAL19* transcript isoforms that were assembled with these methods was poor. For each *DAL19* transcript isoform and each assembler, we calculated a similarity score. This score consisted of the number of base matches of the assembled transcript with the longest match to a *DAL19* transcript isoform divided by the number of bases in that *DAL19* transcript isoform. Trinity only assembled *DAL19_αψγ* and *DAL19_αψδ* with more than 90% similarity, and Oases only assembled *DAL19_βψγ* with more than 90% similarity (Table 2). Therefore, we developed a novel approach based on naive De Bruijn graph traversal coupled with kallisto (Bray et al., 2016) to reconstruct likely transcript isoforms containing 47-mers in the *DAL19* core region. Our method reconstructed *DAL19_αψγ, DAL19_αψδ*, and *DAL19_βψδ* at more than 90% similarity.

**Table 2:** Assembled transcripts with best match with known full-length *DAL19* transcript isoforms.

*DAL19 transcript*	*Length of DAL19 transcript*	*Assembly*	*Similarity*	*CIGAR*
*DAL19_αψδ*	842	Oases	0.77	103S305M1D347 M405S
*DAL19_αψγ*	813	Oases	0.87	103S13M1I291M1 D241M105I 267M139S
*DAL19_βψδ*	1095	Oases	0.80	872M405S
*DAL19_βψγ*	1067	Oases	0.97	1033M139S
*DAL19_αψδ*	842	Trinity	0.92	267S774M62S
*DAL19_αψγ*	813	Trinity	0.92	267S746M142S
*DAL19_βψδ*	1095	Trinity	0.68	274S767M62S
*DAL19_βψγ*	1067	Trinity	0.39	7M1I1M1I23M2I 12M4D234M2I3M 1D7M1D16M1D2M1I 209M310S
*DAL19_αψδ*	842	cortexpy/kallisto	1.00	110S842M52S
*DAL19_αψγ*	813	cortexpy/kallisto	1.00	110S13M1I800M139S
*DAL19_βψδ*	1095	cortexpy/kallisto	0.92	1008M52S
*DAL19_βψγ*	1067	cortexpy/kallisto	0.45	177S31M6D41M2D4 M1D4M5I2M4I3M1I8M 2I10M3I5M3I2M1I35 M2I8M2D1M2D4 72M524S
*DAL19_αψδ*	842	Nine bud samples	1.00	81S842M52S
*DAL19_αψγ*	813	Nine bud samples	1.00	81S13M1I800M69S
*DAL19_βψδ*	1095	Nine bud samples	0.96	1050M12S
*DAL19_βψγ*	1067	Nine bud samples	0.86	920M


*For each *DAL19* transcript and each assembly the shortest assembled transcript with the longest sequence match to the *DAL19* transcript is reported. **Assembly**: name of assembly tool that generated the transcripts (see Assembly, Evaluation, and Visualization of DAL19 Transcripts). **Similarity**: number of base matches to the *DAL19* transcript divided by the number of bases in the *DAL19* transcript. **CIGAR**: CIGAR string of transcript alignment to the *DAL19* transcript in SAMv1 format.*

From the PacBio Iso-Seq data, we found at least one CCS read that was consistent over its entire length with only one of the transcripts *DAL19_αψγ, DAL19_αψδ, DAL19_βψγ*, or *DAL19_βψδ* (Figure 2). We also found CCS reads that mapped to the *ψ* region, but not to the α or β region. Although these reads could be evidence of *DAL19* transcripts without an *α* or *β* region, loss of 5′ ends due to the PacBio Iso-Seq library prep has been observed previously (Sharon et al., 2013; Gordon et al., 2015), and at least one read contained *k*-mers that spanned the junction of *DAL19_α* and *DAL19_ψ*.

### The Different *DAL19* Isoforms Have Distinct Expression Profiles in Early Bud Meristems

From previous experiments, we know that *DAL19* activity is upregulated in both needles and apical buds of cone-initiating *acrocona* shoots (Uddenberg et al., 2013). A reexamination using transcript-specific primers that amplify at similar efficiency indicates that it is the *DAL19_αψδ* isoform that dominates in cone-setting *acrocona* shoots (Supplementary Table S5).

**FIGURE 3:**
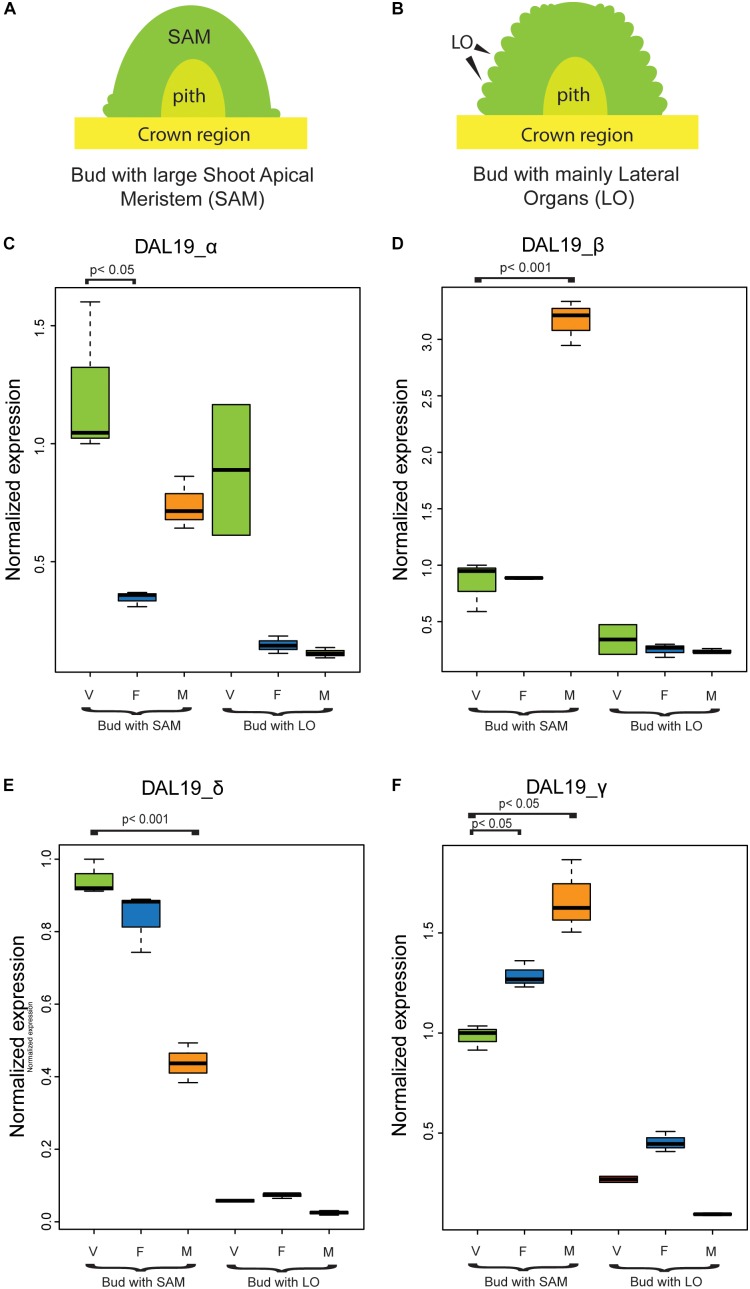
Normalized expression of *DAL19* isoforms assayed by qRT-PCR. **(A)** Schematic representation of a bud with a large Shoot Apical Meristem (SAM) commonly found during late July or early August. **(B)** Schematic representation of a bud in late August or early September, when lateral organ formation is ongoing. **(C–F)** Normalized expression of the *DAL19* isoforms with α **(C)**, β **(D)**, δ **(E)**, and γ **(F)** exons. V, vegetative; 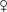, female; 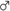, male.

To substantiate these findings and to study if the different DAL19 isoforms are also differentially regulated in wild-type *Picea abies*, we analyzed their expression in buds of different identities (i.e., vegetative, male and female identity) using quantitative Real-Time PCR (qRT-PCR) (Figure 3). Templates for the qRT-PCR experiments were vegetative, female, and male buds collected during early bud development when most of the bud only consists of a large shoot-apical meristem (Figure 3A). As control samples, we included bud samples from a later phase of lateral organ formation (Figure 3B). *DAL19* isoform-specific primers with similar amplification efficiencies were used to assay relative transcript abundance in the different samples (Supplementary Figure S4). With the exception of transcripts with 5′ region *DAL19_α* in vegetative and male samples, all transcripts amplified at significantly higher levels in the early-phase meristems relative to buds collected at the later lateral-organ-formation-phase (Figures 3C–F). Relative to vegetative meristems, the isoforms containing the 5′ region *DAL19_β* amplified at a significantly higher level in male meristems (Figure 3D), whereas the isoforms containing the 5′ region *DAL19_α* amplified at significantly lower levels in female buds (Figure 3C). The isoforms containing the 3′ region *DAL19_δ* were significantly down-regulated in male meristems compared to vegetative meristems, whereas the isoforms containing the 3′ region *DAL19_γ* were significantly up-regulated in both female and male meristems (Figures 3E, F). Hence, by comparing ΔΔCT values in male and female buds relative to vegetative meristems, isoforms with *DAL19_β* and *DAL19_γ* appear to be upregulated in male buds, possibly reflecting an up-regulation of the *DAL19_βψγ* transcript in male meristems.

**FIGURE 4:**
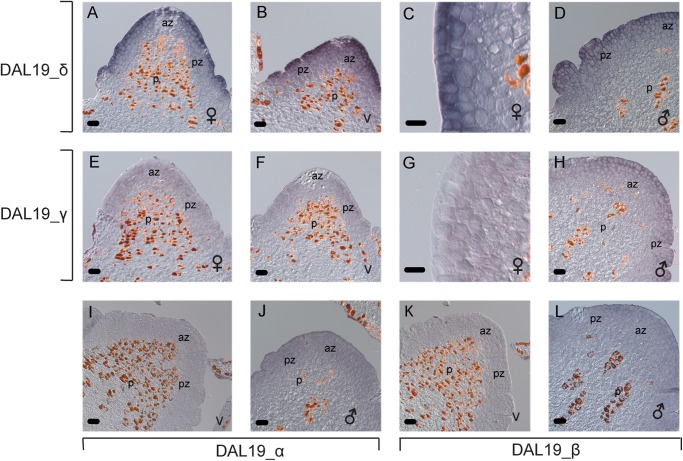
*In situ* hybridization using short *DAL19* isoform specific LNA probes. Micrographs of longitudinal sections of bud meristems, v, vegetative; 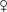, female; 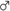, male; *p*, central pith; az, apical zone of the shoot apical meristem; pz, peripheral zone of the meristem. Micrographs **(A–D)** are hybridized with an LNA-probe that specifically binds to *DAL19_δ*-containing transcripts. Sections in micrographs **(E–H)** are hybridized with a *DAL19_γ* probe, **(I,J)** with a *DAL19_α* probe, and **(K,L)** with a *DAL19_β* probe. Size bar = 50 μm in **(A,B,D–F,H–L)**, 20 μm in **(C,G)**.

To test if the differences found in the qRT-PCR experiments are reflected in a spatial distribution of the *DAL19* isoforms in the early bud meristems, we conducted mRNA *in situ* hybridization experiments using isoform specific LNA probes hybridized against longitudinal sections of female, vegetative and male buds. Using a *DAL19_δ* specific probe we detected distinct hybridization signals in the epidermal cell layer of female and vegetative buds, and emerging lateral organs (Figures 4A–C). In male buds at a similar stage, the hybridization signal was less distinct in the epidermal layer and more evenly distributed into the underlying cell layers in the bud meristem (Figure 4D).

We detected a weak hybridization signal, almost complementary to that of *DAL19_δ*, in sections of female and vegetative buds hybridized with *DAL19_γ*-specific LNA-probes (Figures 4E–G). In longitudinal sections of female and vegetative buds, *DAL19_γ* hybridization was reduced or absent in the epidermal cell layer, whereas the signal was stronger in the underlying cell layers of the bud meristems (Figure 4G). In male buds, the signal from *DAL19_γ* LNA-probes matched that of *DAL19_δ* (Figure 4H). LNA-probes directed toward the 5′ isoforms containing *DAL19_α* or *DAL19_β* showed a considerably weaker signal that is difficult to distinguish from the background (Figures 4I,J). Reliable signal of *DAL19_β* was only detected in male meristems, in a pattern that matched those of *DAL19_δ* and *DAL19_γ* (Figures 4D,H,L). *DAL19_α* signals were evenly distributed throughout the whole meristematic region of the bud but were lower in the central pith (Figure 4). LNA probes directed toward *Histone H2A*, included as positive experimental control, gave a characteristic patchy signal in dividing cells (Supplementary Figure S5). Taken together, this demonstrates that distinct *DAL19* isoforms are active in bud meristems of different identity and that cell-type-specific distribution of at least the isoforms containing *δ* or *γ* can occur within one bud meristem.

### Our Novel Assembly Approach Identifies 1,084 Putative MADS-Box Transcripts From Short-Read Sequencing

To assess if other novel MADS-box transcripts exist that are similar to the transcripts we discovered in *DAL19*, we applied our assembly method to RNA-seq data from nine meristematic bud samples to reconstruct likely transcript isoforms (see Assembly and Filtering of MADS-Box Transcripts in Section “Materials and Methods”). We merged assembled transcripts from all bud samples, annotated them with predicted protein domains, and mapped them back to MADS-box core regions. This resulted in 1,084 unique assembled transcripts, of which 1,073 could be annotated with at least one predicted protein domain, and of which 1,040 further mapped to a MADS-box core region. We manually curated these 1,040 assembled transcripts and performed multiple sequence alignment to arrive at 933 likely transcript isoforms. We present single nucleotide polymorphisms (SNPs), indels, and usage of alternate exons in Table 3.

**Table 3:** Summary of transcripts aligned to MADS-core regions.

*Core name*	*Associated MADS_MA*	*Tot. Number of transcripts*	*InDels*	*Alternate 5′ exon*	*Alternate 3′ exon*	*Short transcripts*
*DAL1*	MA_453949	86	Yes	No	Yes, MA_369170 or MA_128164	Yes_only_MADS
*DAL3*	MA_141872, MA_10256834,	55	Yes	Yes	No	Yes_only_MADS due to frame shift
*DAL3_like*	MA_19007, MA_32676	33				
*DAL4*	MA_35712, MA_101463	139	No	Yes	No	Yes-3′ frame shift
*DAL10*	MA_15122	84	Yes	No	No	Yes-5′ frame shift
*DAL11*	MA_18048	22	Yes	No	No	Yes-3′ frame shift
*DAL12*	MA_13933	28	Yes	No	No	Yes-3′ frame shift
*DAL13*	MA_130755	25	Yes	No	No	No
*DAL13_Like_a*	MA_2191711	4	Yes	No	No	No
*DAL13_like_b*	MA_8198323	3	Yes	No	Yes	Yes_only_MADS
*DAL19*	MA_16120, MA_162822, MA_329880,	90	Yes	Yes	Yes	Yes_only_K-domain
*DAL21*	MA_126898	21	Yes	No	No	Yes- 3′ frame shift
*DAL19_like_a/DAL31*	MA_7272891	22	No	No	No	No
*DAL32*	MA_7858089, MA_138440	59	Yes	Yes	Yes, MA_G76145 or MA_109338	Yes_MA_576145
*DAL33*	MA_10079394, MA_276701	123	Yes	Yes	No	Yes-only MADS
*DAL34*						
*DAL35*	MA_9284799	20	No	No	No	No
*DAL37*	MA_18942	20	No	No	No	No
*DAL38*	MA_20180	12	No	No	Yes, MA_88284 or MA-63649	
*DAL39*	MA_94049	25	No	No	No	No
*DAL40/JTL*	MA_10432602	14	No	No	No	No
*DAL41*	MA_57186	26	No	No	No	No
*DAL5*	MA_32019	3	No	No	No	No
*DAL9*	MA_6279308	19	No	No	No	No
*Sum*		933				


*Core name: The name of the MADS-box core sequence used in the analysis. Associated MADS_MA: The identifier of the scaffold harboring the first exon of the assembled transcripts. Total Number of transcripts: Number of transcripts associated with a specific isoform. InDels: Indication if InDels occurs among the aligned transcripts. Alternate 5′ exon: Indication if mutually exclusive 5′ exons were detected. Alternate 3′ exons: Indication if mutually exclusive 3′ exons were detected. Short transcripts: Indication if the gene has an alternative splice site selection at the 3′ or 5′ end of exons, rendering short transcripts.*

We identified multiple isoforms of *DAL19*, which is consistent with the results presented in Section “Mapping of DAL19 Transcripts to Genomic Assemblies Provides Support That These Transcripts Are Isoforms.” We assembled transcripts that matched the cloned and confirmed *DAL19* transcripts *αψγ, αψδ, βψγ*, and *βψδ* with a similarity of 0.999, 0.998, 0.96, and 0.86, respectively (Table 2). The CIGAR strings for the alignments of the assembled transcripts to these *DAL19* transcripts (Table 2) indicated that the assembled transcript for βψδ was missing 147 bases, and that the assembled transcripts for the other three *DAL19* transcripts contained fewer than 100 extra bases on the 5′ or 3′ end of the transcript. Furthermore, we detected one additional *DAL19* transcript. This transcript contained a MADS-box that mapped to sequence scaffold MA_162822 in the *P. abies* genome assembly (V1.0).

As for *DAL19*, we also identified the use of alternate 5′ exons for the core sequences of *DAL3, DAL3_like, DAL4, DAL32*, and *DAL33*. These first exon sequences mapped to distinct scaffolds in the *P. abies* genome assembly (V1.0) (Table 3, column 2) and nucleotide searches against the NCBI Conserved Domain Database (SDD) indicated that these exons all encode MADS-domains. However, the transcript isoform of *DAL33* that mapped to the genomic scaffold MA_10079394 differed substantially on the amino acid level, albeit not on the nucleotide level, from other known MADS-domains and lacked most conserved MADS-domain signature motifs such as for example the IKRIENS, RQVT, and the KKYELS motifs (Supplementary Figure S6A).

Apart from for *DAL19*, we identified the use of alternate 3′ exons for the core sequences of the gene *DAL1* (Supplementary Figure S6B). As with *DAL19*, the usage of an alternate 3′ exon resulted in a premature stop codon and loss of the C-terminal domain, which harbors the signature motif of AGL6-like genes (DCEPTLQIGY). We also identified transcripts with a premature stop codon, often occurring shortly after the first exon, in *DAL3, DAL4, DAL12, DAL21, DAL32, DAL33* and *DAL38*. In the case of *DAL13*, we assembled three transcripts with several SNPs that were distributed over the entire ORF. In BLAST searches against the *P. abies* genome V1.0 these three *DAL13-*related transcripts mapped to different sequence scaffolds indicating that these transcripts were transcribed from different genes.

To provide independent evidence for the occurrence of different MADS-box gene isoforms, we mapped CCS reads to the assembled transcript isoforms and aggregated them by MADS-box core sequence. The number of CCS reads that only mapped to transcripts associated with a single MADS-box core sequence are presented in Table 1. The CCS reads were consistent with assembled transcript isoforms *DAL1, DAL3, DAL3_like_a, DAL4, DAL9, DAL19, DAL31, DAL32, DAL33, DAL35, DAL38, DAL37, DAL40/JTL*, and *DAL41* (Table 1).

### 174 Transcripts Are Differentially Expressed Across Three Bud Types

We assessed the differential expression of all assembled transcripts (1,084) across two male, three female, and three vegetative meristematic bud samples using a quasi-likelihood *F*-test (McCarthy et al., 2012) implemented in the edgeR package (Robinson et al., 2010). Hundred and seventy four transcripts were differentially expressed across all three groups at an FDR of 0.1 (Supplementary Table S6). We had previously assigned 152 of the 174 differentially expressed transcripts to 28 putative MADS-box genes (Table 1). The remaining ten putative MADS-box genes did not have any differentially expressed transcripts assigned. Of the 16 putative MADS-box genes for which there was evidence of expression from CCS reads, 14 genes had differentially expressed transcripts assigned.

### All MADS-Box Genes Using Alternate First Exons Fall Into a Common Clade in Phylogenetic Analysis

To examine to what extent the usage of alternate exons in different MADS-box gene isoforms influences the position of MADS-box gene isoforms in the phylogenetic tree, maximum parsimony analyses were performed. In a first analysis, the nucleotide sequence spanning the open reading frame (ORF) of transcripts representing the identified MADS-box gene isoforms were aligned to MADS-box genes from *Picea abies, Pinus radiata, Arabidopsis thaliana*, and *Lycopersicon esculentum*, Supplementary Figure S7A. In a second analysis, only the MADS-box was used to determine the phylogenetic relationship (Supplementary Figure S7B).

In both analyses, and in agreement with previous published analyses, functionally related angiosperm MADS-box genes form monophyletic clades that often also have a gymnosperm sister-clade. For instance, *DAL2* from *Picea abies* and PrMADS1 grouped close to the *AGAMOUS*-clade as reported by (Tandre et al., 1995) and *DAL11-13* grouped with the angiosperm clade harboring AP3 and PI as reported by (Sundstrom et al., 1999). In the first analysis, all *Picea abies* genes harboring alternate MADS-box sequences, e.g., *DAL3, DAL4, DAL19, DAL32*, and *DAL33*, fell into a common clade that formed a sister clade to the angiosperm TM3 clade. As expected, transcript isoforms belonging to the same MADS-box core sequence grouped together. In the second analysis, which was based on only the nucleotide sequences encoding MADS-domains, the majority of the isoforms still grouped in the same sub-clade. However, the internal relationships between isoforms and other MADS-box genes changed, and transcript isoforms assigned to the same core region were split up in different sub-clades. For example, in the first analysis, *DAL3* transcripts number 160 and 432 formed a well-supported subclade (bootstrap value 99) together with the transcript for *DAL3* deposited in Genbank (XY6654356). In the second analysis the *DAL3* sub-clade was split into two distinct sub-clades of which one grouped together with the transcript *DAL3 Like 966*. In addition, there was lack of support for the position of the *DAL33* transcript number 162 in the larger sub-clade of gymnosperm genes that formed a sister-clade to the *TM3*-subclade.

Apart from *DAL19*, assembled isoforms that used mutually exclusive exons in the 3′ region were detected for the gene *DAL1*. In the phylogenetic reconstruction, *DAL1* and its corresponding isoforms did not group in the same sub-clade as the other genes with reported 5′ isoforms. *DAL1, DAL14*, and the isoforms represented by transcripts number 580 and 802 instead formed a sister-clade to angiosperm *AGL6/AGL2-like* genes.

## Discussion

A multi-exon gene may be spliced into numerous variants through exon skipping, exon mutual exclusion, intron retention, or alternative splice site selection at the 3′ or 5′ end of exons (A3 or A5, and as reviewed by Reddy et al., 2013). Our analysis shows that all forms of alternative splicing occur in the MADS-box gene family in *P. abies*. Notably, the use of mutually exclusive first exons occurs strikingly often. We identified usage of mutually exclusive first exons in the genes *DAL3, DAL3_like, DAL4, DAL19, DAL32*, and *DAL33.* In these genes, the first exon encodes the DNA-binding MADS-domain. It is the MADS-domain that is responsible for the DNA-binding properties of the proteins and it has been demonstrated that this domain interacts with *cis*-regulatory DNA elements called CArG-boxes (Riechmann et al., 1996). Recently, it has also been demonstrated that small changes in the MADS-box amino acid composition might influence the affinity of the MADS-box to different CArG-box sequences (Smaczniak et al., 2017). The MADS-box domain of isoforms containing *DAL19_α* or *DAL19_β* differs in several amino acids: Two of the amino acids are in the highly conserved IKRIENS-motif and five amino acids differ in the region of the MADS-domain, which, according to structural models, is thought to encode β-sheets (Supplementary Figure S6A). A similar frequency of amino acid changes is also found in the MADS-domains of DAL3, DAL3_like, DAL4, DAL19 and DAL32 (Supplementary Figure S6A). We hypothesize that the isoforms of these genes have different affinity to different CArG-box sequences and may thereby regulate different sets of target genes. In the gene *DAL33*, one of the isoforms has diverged considerably and has accumulated substitutions primarily in the first and second positions of the triplet codons that encode the MADS-domain signature motifs, indicating that this isoform of *DAL33* may confer a change or complete loss of DNA binding properties to the DAL33 protein.

Apart from changes in DNA-binding properties, usage of mutually exclusive first exons may also confer different transcriptional activity. In fact, (Li et al., 2007) argue that mutually exclusive usage of first exons may constitute a distinct form of mutual exon exclusion because it also implies that the isoforms are transcribed from different promoters. In line with this, we detected differential expression of the isoforms containing *DAL19_α* or *DAL19_β* across female, vegetative, and male bud samples.

Several lines of evidence suggest that MADS-domain transcription factors form homo or hetero dimers, reviewed by Gramzow and Theissen (2010). In addition, both angiosperm and gymnosperm MADS-domain transcription factors active during reproductive development form multimeric complexes that have the ability to bind several CArG-boxes through DNA-looping (Kaufmann et al., 2005). Hence, the activity of a MADS-domain protein is determined both by its DNA-binding properties and its ability to interact with other MADS-domain proteins and associated proteins. Structural characterization of the intervening (I) and keratin-like (K) domains of SEPPALATA3 (SEP3) from *Arabidopsis thaliana* has demonstrated that the domains form two amphipathic alpha helices and that regularly spaced hydrophobic residues in those two helices are important for dimerization and for the formation of higher order tetramer complexes (Puranik et al., 2014). In the DAL19 protein, the K-domain and the intervening region corresponds to the core region (*ψ*) defined here. We found no evidence of usage of mutually exclusive exons in the K-domain of the *P. abies* MADS-domain proteins although occasional retention of introns and alternative splice site selection could be observed. This suggests that alternative splicing does not primarily affect protein dimerization properties. This has implications for the interpretation of functional relevance of the short mature mRNA transcripts that lack DNA-binding MADS-domain i.e., *DAL19_ψα* or *DAL19_ψβ*. It is possible that transcription of a short transcript that has the ability to interact with other MADS-domain proteins but lacks the DNA-binding domain, may in fact act as a dominant negative protein.

We also identified use of mutually exclusive exons in the 3′ region of the genes *DAL1*, and *DAL19*. In both genes, usage of an alternate 3′ exon leads to a shorter mature mRNA transcript that lacks the C-terminal signature motifs (Supplementary Figure S6B). *DAL19_δ* harbors the signature motif (EVETQL) commonly found in TM3-like genes, whereas transcripts ending with the *DAL19_γ* exon yield a protein with a premature stop codon. Similarly, transcripts ending with *DAL1_α* harbor the AGL6-like motif (DCEPTLQIGY) whereas the usage of an alternate 3′ C-terminal exon in *DAL1_β* leads to a pre-mature stop codon directly after the K-domain. It has been demonstrated that the C-terminal of MADS-box genes are critical for functional specificity (Lamb and Irish, 2003) as the C-terminal may harbor activation domains or allow interactions with specific proteins (Litt and Irish, 2003). As judged by sequence comparison with the structurally characterized SEP3-protein (Puranik et al., 2014), the long protein isoforms of DAL1 and DAL19 have retained conserved hydrophobic residues in the last part of the K-domain that are important for dimerization and tetramerization. The short versions have retained all residues of importance for dimerization, but due to the usage of alternate 3′ exons they lack part of the hydrophobic residues that have been shown to be of importance for tetramerization in SEP3. Hence, provided that the short transcript isoforms of *DAL1* and *DAL19* are translated into proteins, the resulting proteins may have retained their ability to dimerize but could have lost their ability to form higher order complexes and may in fact work as dominant negative proteins.

The occurrence of alternatively spliced *DAL19* and *DAL1* isoforms with premature stop codons is analogous to the MADS-box gene *FLOWERING LOCUS M* (*FLM*) in *A. thaliana*, which in its active form acts as a repressor of flower development (Pose et al., 2013). An increase in ambient temperature leads to alternative splicing of the FLM transcripts and the formation of a premature stop, which in turn triggers nonsense mediated decay (Sureshkumar et al., 2016; Capovilla et al., 2017). In this case, alternative splicing influences the amount of active protein in a specific cell or tissue. In our data, isoforms containing *DAL19_δ* were expressed at high levels in the epidermal layer of vegetative and female bud meristems, whereas the isoforms containing *DAL19_y* showed a complementary expression pattern in the same meristem. This is strong evidence that cell-specific splicing may occur within a single meristem. Alternative splicing may down-regulate the amount of active protein in a specific cell through nonsense mediated decay or expression of dominant negative forms of the protein. This may contribute to the establishment of sharp boundaries within a meristem between cells that express the active form of the protein and cells that express the inactive or dominant negative form of the protein.

Furthermore, apart from use of mutually exclusive first or last exons, we also observe use of alternative 5′ and 3′ splice site selection in *DAL3, DAL4, DAL12, DAL21, DAL32, DAL4, DAL3, DAL33*, and *DAL38*. Studies in the model plant *Arabidopsis thaliana*, and other angiosperm species, indicate that this form of alternative splicing is more prevalent than the use of mutually exclusive exons (Severing et al., 2012; Zhang et al., 2015; Luo et al., 2017; Verhage et al., 2017). Among the *P. abies* MADS-box genes, these alternative splice sites often result in frame shifts and premature stop-codons shortly after the MADS-box region. This suggests that several MADS-box genes may be translated into both full-length proteins and micro-proteins.

Taken together, use of mutually exclusive first exons provides a means to express MADS-box genes from different promoters in a tissue or bud specific manner. The occurrence of different amino acids in the MADS-domains may confer varying DNA-binding properties to the resulting MADS proteins. This may affect the selection of down-stream target genes and may thereby change the regulation of bud development. The use of mutually exclusive last exons or alternative splice site selection provides a means to either produce proteins with different function in a cell-specific manner, or to establish sharp boundaries between an active and an inactive isoform within a single meristem.

We also present a novel approach to transcriptome assembly. We used this approach to assemble *DAL19* transcript isoforms that match the cloned and confirmed *DAL19* isoforms *αψγ, αψδ*, and *βψγ*, except for the 5′ ends of the α and β regions and the 3′ ends of the δ and γ regions. However, where the assembled and cloned transcripts diverged, CCS reads supported the assembled and not the cloned transcripts (dark green, dark blue, and dark red colors in Supplementary Figures 2A,B). It is likely that the cloned transcripts were shorter at the 3′ ends because the 5′/3′ RACE approach used to clone these transcripts is not guaranteed to clone the full length of the 3′ transcript end. The divergence on the 5′ end of the transcripts may be a rare variant in the *P. abies* reference.

We furthermore used our assembly approach on nine other bud samples to show that *P. abies* expresses hundreds of transcript isoforms containing one of 38 MADS-box core sequences. We found 933 plausible transcripts of which 152 were differentially expressed across bud types, and of which the majority clustered in an expected manner with known DAL transcripts in phylogenetic analyses of full-length transcripts and of only the MADS-box region. A minority of plausible MADS-box transcripts displayed different clustering between the two phylogenetic analyses. This could be due to assembly errors or it could reflect real gene fusions in the evolutionary history of these transcripts.

Our assembly approach appears to have high sensitivity to transcript isoforms of the MADS-box gene family in *P. abies*. However, further work is needed to establish the false positive rate of our method, how this method performs more generally for the *P. abies* transcriptome, and how it can be used for assembling the transcriptomes of other organisms. Our method generates a candidate transcript for every possible 5′ to 3′ path through a De Bruijn graph representation of RNA-seq reads, and it relies on kallisto to filter these candidate transcripts down to a reasonable number. Unfortunately, the number of candidate transcripts generated scales exponentially with the number of branches between any incoming and outgoing tip in the De Bruijn graph. Therefore, genes with more splice variants or sequence polymorphisms could cause the number of candidate transcripts to grow to a size that kallisto cannot manage. Our approach does not handle cycles in the graph, although links as described by Turner et al. (2018) could be used to traverse small cycles. Finally, our approach cannot detect truncated transcripts together with full-length transcripts, as candidate transcripts may only start on incoming tips of the De Bruijn graph. However, allowing candidate transcripts to also start from *k*-mers with a large coverage increase compared to their incoming neighbor *k*-mers might allow the detection of truncated transcripts.

Phylogenetic reconstructions of the MADS-box gene family have shown that functionally related genes group together in monophyletic clades (see e.g., Becker and Theissen, 2003 and references within). Based on genome-wide analyses and transcriptome data it has also been suggested that in gymnosperms, MADS-box genes orthologous to *DAL19* have undergone a series of duplication events leading to a rapid expansion in the number of genes and the formation of a *DAL19*-clade in several gymnosperm lineages (Gramzow et al., 2014; Chen et al., 2017).

Based on the phylogenetic reconstruction of the MADS-box gene family and their transcript isoforms, all *P. abies* MADS-box genes that display alternative splicing of mutually exclusive first exons grouped together in the *DAL19*-clade. Hence, the observed complexity in this clade may not only be due to duplication events but also due to alternative splicing and usage of mutually exclusive first exons. We also note that phylogenies based solely on the conserved MADS-box may lead to an overestimation of the number of genes and changes in the tree topology, which in turn could influence the interpretation of the phylogenetic relationships between different MADS-box genes. Functional characterization of the angiosperm genes within the *TM3-like* clade has demonstrated that several genes are involved in the transition from vegetative to reproductive growth. We hypothesize that increased complexity in terms of number of genes and usage of transcript isoforms in the gymnosperm sister-clade to *TM3-like* genes reflects a complex genetic regulation of vegetative to reproductive phase change and cone-setting in conifers and other gymnosperms. This regulation possibly involves responses to environmental factors such as ambient temperature or daylight that may influence splicing and the transcription of different transcript isoforms. Temperature is, in fact, used as a predictor of cone initiation in *P. abies* (Lindgren et al., 1977), and we hypothesize that alternative splicing is one of the molecular mechanisms employed by the tree to determine whether or not to produce cones.

## Author Contributions

SA collected the plant material, performed qPCR, and discovered putative MADS-box core sequences. SA and VN prepared the RNA and performed the cloning. ON, OE, and JS provided funding for the Illumina sequencing. ON provided funding for the PacBio sequencing. ND, VN, and NS pre-processed the short-read and long-read sequencing data. WK developed the assembly method and performed the assembly, filtering, mapping, and differential expression analysis of transcripts. JS curated the transcript isoforms and performed the phylogenetic analysis. SA and JS performed *in situ* hybridization. JS supervised SA and OE supervised WK. JS and ON supervised VN. SA, WK, and JS wrote substantial portions of the manuscript. SA, WK, and JS designed the study. All authors edited the manuscript.

## Conflict of Interest Statement

The authors declare that the research was conducted in the absence of any commercial or financial relationships that could be construed as a potential conflict of interest.

## Footnotes

1 congenie.org

2 https://github.com/samtools/hts-specs

3 https://github.com/winni2k/cortexpy

4 http://www.geneious.com

5 http://congenie.org

## Supplementary Material

All sequencing data associated with this paper can be obtained from the European Nucleotide Archive with the accession numbers PRJEB27247 and ERP109307.

## References

[B1] Abdel-Ghany S. E. Hamilton M. Jacobi J. L. Ngam P. Devitt N. Schilkey F. (2016). A survey of the sorghum transcriptome using single-molecule long reads. *Nat. Commun.* 7:11706. 10.1038/ncomms11706 27339290 PMC4931028

[B2] Azevedo H. Lino-Neto T. Tavares R. M. (2003). An improved method for high-quality RNA isolation from needles of adult maritime pine trees. *Plant Mol. Biol. Rep.* 21 333–338. 10.1007/BF02772582

[B3] Becker A. Theissen G. (2003). The major clades of MADS-box genes and their role in the development and evolution of flowering plants. *Mol. Phylogenet. Evol.* 29 464–489. 10.1016/S1055-7903(03)00207-0 14615187

[B4] Benjamini Y. Hochberg Y. (1995). Controlling the false discovery rate : a practical and powerful approach to multiple testing. *J. R. Stat. Soc. Ser. B* 57 289–300.

[B5] Bolger A. M. Lohse M. Usadel B. (2014). Trimmomatic: a flexible trimmer for Illumina sequence data. *Bioinformatics* 30 2114–2120. 10.1093/bioinformatics/btu170 24695404 PMC4103590

[B6] Bray N. L. Pimentel H. Melsted P. Pachter L. (2016). Near-optimal probabilistic RNA-seq quantification. *Nat. Biotechnol.* 34 525–527. 10.1038/nbt.3519 27043002

[B7] Capovilla G. Symeonidi E. Wu R. Schmid M. (2017). Contribution of major FLM isoforms to temperature-dependent flowering in *Arabidopsis thaliana*. *J. Exp. Bot.* 68 5117–5127. 10.1093/jxb/erx328 29036339 PMC5853260

[B8] Carlsbecker A. Sundstrom J. F. Englund M. Uddenberg D. Izquierdo L. Kvarnheden A. (2013). Molecular control of normal and acrocona mutant seed cone development in Norway spruce (*Picea abies*) and the evolution of conifer ovule-bearing organs. *New Phytol.* 200 261–275. 10.1111/nph.12360 23772833

[B9] Chen F. Zhang X. Liu X. Zhang L. (2017). Evolutionary analysis of MIKC^c^-type MADS-box genes in gymnosperms and angiosperms. *Front. Plant Sci.* 8:895. 10.3389/fpls.2017.00895 28611810 PMC5447709

[B10] Conesa A. Madrigal P. Tarazona S. Gomez-Cabrero D. Cervera A. Mcpherson A. (2016). A survey of best practices for RNA-seq data analysis. *Genome Biol.* 17:13. 10.1186/s13059-016-0881-8 26813401 PMC4728800

[B11] Dorca-Fornell C. Gregis V. Grandi V. Coupland G. Colombo L. Kater M. M. (2011). The Arabidopsis SOC1-like genes AGL42, AGL71 and AGL72 promote flowering in the shoot apical and axillary meristems. *Plant J.* 67 1006–1017. 10.1111/j.1365-313X.2011.04653.x 21609362

[B12] Dwyer T. Koren Y. Marriott K. (2006). IPSEP-COLA: an incremental procedure for separation constraint layout of graphs. *IEEE Trans. Vis. Comput. Graph* 12 821–828. 10.1109/TVCG.2006.156 17080805

[B13] Gordon S. P. Tseng E. Salamov A. Zhang J. Meng X. Zhao Z. (2015). Widespread polycistronic transcripts in fungi revealed by single-molecule mRNA sequencing. *PLoS One* 10:e0132628. 10.1371/journal.pone.0132628 26177194 PMC4503453

[B14] Grabherr M. G. Haas B. J. Yassour M. Levin J. Z. Thompson D. A. Amit I. (2011). Full-length transcriptome assembly from RNA-Seq data without a reference genome. *Nat. Biotechnol.* 29 644–652. 10.1038/nbt.1883 21572440 PMC3571712

[B15] Gramzow L. Theissen G. (2010). A hitchhiker’s guide to the MADS world of plants. *Genome Biol.* 11:214. 10.1186/gb-2010-11-6-214 20587009 PMC2911102

[B16] Gramzow L. Weilandt L. Theissen G. (2014). MADS goes genomic in conifers: towards determining the ancestral set of MADS-box genes in seed plants. *Ann. Bot.* 114 1407–1429. 10.1093/aob/mcu066 24854168 PMC4204780

[B17] Hannerz M. Sundström J. F. (2006). “I år blommar granen”, in: *PlantAktuellt*, ed. Valinger E. (Uppsala: Skogforsk).

[B18] Hoang N. V. Furtado A. Mason P. J. Marquardt A. Kasirajan L. Thirugnanasambandam P. P. (2017). A survey of the complex transcriptome from the highly polyploid sugarcane genome using full-length isoform sequencing and de novo assembly from short read sequencing. *BMC Genomics* 18:395. 10.1186/s12864-017-3757-8 28532419 PMC5440902

[B19] Jasaitiene D. Valiukeviciene S. Linkeviciute G. Raisutis R. Jasiuniene E. Kazys R. (2011). Principles of high-frequency ultrasonography for investigation of skin pathology. *J. Eur. Acad. Dermatol. Venereol.* 25 375–382. 10.1111/j.1468-3083.2010.03837.x 20849441

[B20] Karlgren A. Carlsson J. Gyllenstrand N. Lagercrantz U. Sundstrom J. F. (2009). Non-radioactive in situ hybridization protocol applicable for Norway spruce and a range of plant species. *J. Vis. Exp.* 26:e1205. 10.3791/1205 19377440 PMC3148633

[B21] Kaufmann K. Melzer R. Theissen G. (2005). MIKC-type MADS-domain proteins: structural modularity, protein interactions and network evolution in land plants. *Gene* 347 183–198. 10.1016/j.gene.2004.12.014 15777618

[B22] Kopylova E. Noé L. Touzet H. (2012). SortMeRNA: fast and accurate filtering of ribosomal RNAs in metatranscriptomic data. *Bioinformatics* 28 3211–3217. 10.1093/bioinformatics/bts611 23071270

[B23] Krzywinski M. Schein J. Birol I. Connors J. Gascoyne R. Horsman D. (2009). Circos: an information aesthetic for comparative genomics. *Genome Res.* 19 1639–1645. 10.1101/gr.092759.109 19541911 PMC2752132

[B24] Lamb R. S. Irish V. F. (2003). Functional divergence within the APETALA3/PISTILLATA floral homeotic gene lineages. *Proc. Natl. Acad. Sci. U.S.A.* 100 6558–6563. 10.1073/pnas.0631708100 12746493 PMC164485

[B25] Li H. (2013). Aligning sequence reads, clone sequences and assembly contigs with BWA-MEM. *Genomics* 2013 1–3.

[B26] Li H. (2018). Minimap2: pairwise alignment for nucleotide sequences. *Bioinformatics* 34 3094–3100. 10.1093/bioinformatics/bty191 29750242 PMC6137996

[B27] Li Q. Lee J. A. Black D. L. (2007). Neuronal regulation of alternative pre-mRNA splicing. *Nat. Rev. Neurosci.* 8 819–831. 10.1038/nrn2237 17895907

[B28] Li W. Godzik A. (2006). Cd-hit: a fast program for clustering and comparing large sets of protein or nucleotide sequences. *Bioinformatics* 22 1658–1659. 10.1093/bioinformatics/btl158 16731699

[B29] Lindgren K. Ekberg I. Eriksson G. (1977). External factors influencing female flowering in *Picea abies* (L.) Karst. *Stud. For. Suec.* 142 1–53.

[B30] Litt A. Irish V. F. (2003). Duplication and diversification in the APETALA1/FRUITFULL floral homeotic gene lineage: implications for the evolution of floral development. *Genetics* 165 821–833. 14573491 10.1093/genetics/165.2.821 PMC1462802

[B31] Liu X. Mei W. Soltis P. S. Soltis D. E. Barbazuk W. B. (2017). Detecting alternatively spliced transcript isoforms from single-molecule long-read sequences without a reference genome. *Mol. Ecol. Res.* 17 1243–1256. 10.1111/1755-0998.12670 28316149

[B32] Luo X. Xu L. Liang D. Wang Y. Zhang W. Zhu X. (2017). Comparative transcriptomics uncovers alternative splicing and molecular marker development in radish (*Raphanus sativus* L.). *BMC Genomics* 18:505. 10.1186/s12864-017-3874-4 28673249 PMC5496183

[B33] Ma H. Yanofsky M. F. Meyerowitz E. M. (1991). AGL1-AGL6, an Arabidopsis gene family with similarity to floral homeotic and transcription factor genes. *Genes Dev.* 5 484–495. 10.1101/gad.5.3.484 1672119

[B34] Mandadi K. K. Scholthof K. B. G. (2015). Genome-wide analysis of alternative splicing landscapes modulated during plant-virus interactions in *Brachypodium distachyon*. *Plant Cell* 27 71–85. 10.1105/tpc.114.133991 25634987 PMC4330581

[B35] Marchler-Bauer A. Derbyshire M. K. Gonzales N. R. Lu S. Chitsaz F. Geer L. Y. (2015). CDD: NCBI’s conserved domain database. *Nucleic Acids Res.* 43 D222–D226. 10.1093/nar/gku1221 25414356 PMC4383992

[B36] Marquez Y. Brown J. W. Simpson C. Barta A. Kalyna M. (2012). Transcriptome survey reveals increased complexity of the alternative splicing landscape in Arabidopsis. *Genome Res.* 22 1184–1195. 10.1101/gr.134106.111 22391557 PMC3371709

[B37] McCarthy D. J. Chen Y. Smyth G. K. (2012). Differential expression analysis of multifactor RNA-Seq experiments with respect to biological variation. *Nucleic Acids Res.* 40 4288–4297. 10.1093/nar/gks042 22287627 PMC3378882

[B38] Mortazavi A. Williams B. A. Mccue K. Schaeffer L. Wold B. (2008). Mapping and quantifying mammalian transcriptomes by RNA-Seq. *Nat. Methods* 5 621–628. 10.1038/nmeth.1226 18516045 PMC13303166

[B39] Nystedt B. Street N. R. Wetterbom A. Zuccolo A. Lin Y. C. Scofield D. G. (2013). The Norway spruce genome sequence and conifer genome evolution. *Nature* 497 579–584. 10.1038/nature12211 23698360

[B40] O’Maoileidigh D. S. Graciet E. Wellmer F. (2014). Gene networks controlling *Arabidopsis thaliana* flower development. *New Phytol.* 201 16–30. 10.1111/nph.12444 23952532

[B41] Pose D. Verhage L. Ott F. Yant L. Mathieu J. Angenent G. C. (2013). Temperature-dependent regulation of flowering by antagonistic FLM variants. *Nature* 503 414–417. 10.1038/nature12633 24067612

[B42] Puranik S. Acajjaoui S. Conn S. Costa L. Conn V. Vial A. (2014). Structural basis for the oligomerization of the MADS domain transcription factor SEPALLATA3 in Arabidopsis. *Plant Cell* 26 3603–3615. 10.1105/tpc.114.127910 25228343 PMC4213154

[B43] Reddy A. S. Marquez Y. Kalyna M. Barta A. (2013). Complexity of the alternative splicing landscape in plants. *Plant Cell* 25 3657–3683. 10.1105/tpc.113.117523 24179125 PMC3877793

[B44] Riechmann J. L. Wang M. Meyerowitz E. M. (1996). DNA-binding properties of Arabidopsis MADS domain homeotic proteins APETALA1, APETALA3, PISTILLATA and AGAMOUS. *Nucleic Acids Res.* 24 3134–3141. 10.1093/nar/24.16.3134 8774892 PMC146081

[B45] Robertson G. Schein J. Chiu R. Corbett R. Field M. Jackman S. D. (2010). De novo assembly and analysis of RNA-seq data. *Nat. Methods* 7 909–912. 10.1038/nmeth.1517 20935650

[B46] Robinson M. D. Mccarthy D. J. Smyth G. K. (2010). edgeR: a bioconductor package for differential expression analysis of digital gene expression data. *Bioinformatics* 26 139–140. 10.1093/bioinformatics/btp616 19910308 PMC2796818

[B47] Schulz M. H. Zerbino D. R. Vingron M. Birney E. (2012). Oases: Robust de novo RNA-seq assembly across the dynamic range of expression levels. *Bioinformatics* 28 1086–1092. 10.1093/bioinformatics/bts094 22368243 PMC3324515

[B48] Schwarz-Sommer Z. Huijser P. Nacken W. Saedler H. Sommer H. (1990). Genetic control of flower development by homeotic genes in *Antirrhinum majus*. *Science* 250 931–936. 10.1126/science.250.4983.931 17746916

[B49] Severing E. I. Van Dijk A. D. Morabito G. Busscher-Lange J. Immink R. G. Van Ham R. C. (2012). Predicting the impact of alternative splicing on plant MADS domain protein function. *PLoS One* 7:e30524. 10.1371/journal.pone.0030524 22295091 PMC3266260

[B50] Sharon D. Tilgner H. Grubert F. Snyder M. (2013). A single-molecule long-read survey of the human transcriptome. *Nat. Biotechnol.* 31 1009–1014. 10.1038/nbt.2705 24108091 PMC4075632

[B51] Smaczniak C. Angenent G. C. Kaufmann K. (2017). SELEX-Seq: a method to determine DNA binding specificities of plant transcription factors. *Methods Mol. Biol.* 1629 67–82. 10.1007/978-1-4939-7125-1_6 28623580

[B52] Soneson C. Love M. I. Robinson M. D. (2015). Differential analyses for RNA-seq: transcript-level estimates improve gene-level inferences. *F1000Res.* 4:1521. 10.12688/f1000research.7563.1 26925227 PMC4712774

[B53] Steijger T. Abril J. F. Engström P. G. Kokocinski F. Abril J. F. Akerman M. (2013). Assessment of transcript reconstruction methods for RNA-seq. *Nat. Methods* 10 1177–1184. 10.1038/nmeth.2714 24185837 PMC3851240

[B54] Sundell D. Mannapperuma C. Netotea S. Delhomme N. Lin Y. C. Sjdin A. A. (2015). The plant genome integrative explorer resource: plantgenie.org. *New Phytol.* 208 1149–1156. 10.1111/nph.13557 26192091

[B55] Sundstrom J. Carlsbecker A. Svensson M. E. Svenson M. Johanson U. Theissen G. (1999). MADS-box genes active in developing pollen cones of Norway spruce (*Picea abies*) are homologous to the B-class floral homeotic genes in angiosperms. *Dev. Genet.* 25 253–266. 10.1002/(SICI)1520-6408(1999)25:3<253::AID-DVG8>3.0.CO;2-P 10528266

[B56] Sureshkumar S. Dent C. Seleznev A. Tasset C. Balasubramanian S. (2016). Nonsense-mediated mRNA decay modulates FLM-dependent thermosensory flowering response in Arabidopsis. *Nat. Plants* 2:16055. 10.1038/nplants.2016.55 27243649

[B57] Swofford D.L. (2002). *PAUP Phylogenetic Analysis Using Parsimony (and other methods.* Sunderland, MA: Sinauer Associates.

[B58] Tandre K. Albert V. A. Sundas A. Engstrom P. (1995). Conifer homologues to genes that control floral development in angiosperms. *Plant Mol. Biol.* 27 69–78. 10.1007/BF00019179 7865797

[B59] Thatcher S. R. Zhou W. Leonard A. Wang B. B. Beatty M. Zastrow-Hayes G. (2014). Genome-wide analysis of alternative splicing in zea mays: landscape and genetic regulation. *Plant Cell* 26 3472–3487. 10.1105/tpc.114.130773 25248552 PMC4213170

[B60] Turner I. Garimella K. V. Iqbal Z. McVean G. (2018). Integrating long-range connectivity information into de Bruijn graphs. *Bioinformatics* 34 2556–2565. 10.1093/bioinformatics/bty157 29554215 PMC6061703

[B61] Uddenberg D. Reimegard J. Clapham D. Almqvist C. Von Arnold S. Emanuelsson O. (2013). Early cone setting in *Picea abies* acrocona is associated with increased transcriptional activity of a MADS box transcription factor. *Plant Physiol.* 161 813–823. 10.1104/pp.112.207746 23221834 PMC3561021

[B62] Verhage L. Severing E. I. Bucher J. Lammers M. Busscher-Lange J. Bonnema G. (2017). Splicing-related genes are alternatively spliced upon changes in ambient temperatures in plants. *PLoS One* 12:e0172950. 10.1371/journal.pone.0172950 28257507 PMC5336241

[B63] Wang B. Tseng E. Regulski M. Clark T. A. Hon T. Jiao Y. (2016). Unveiling the complexity of the maize transcriptome by single-molecule long-read sequencing. *Nat. Commun.* 7:11708. 10.1038/ncomms11708 27339440 PMC4931018

[B64] Winter K. U. Becker A. Munster T. Kim J. T. Saedler H. Theissen G. (1999). MADS-box genes reveal that gnetophytes are more closely related to conifers than to flowering plants. *Proc. Natl. Acad. Sci. U.S.A.* 96 7342–7347. 10.1073/pnas.96.13.7342 10377416 PMC22087

[B65] Xu Z. Peters R. J. Weirather J. Luo H. Liao B. Zhang X. (2015). Full-length transcriptome sequences and splice variants obtained by a combination of sequencing platforms applied to different root tissues of *Salvia miltiorrhiza* and tanshinone biosynthesis. *Plant J.* 82 951–961. 10.1111/tpj.12865 25912611

[B66] Yeku O. Frohman M. A. (2011). Rapid amplification of cDNA ends (RACE). *Methods Mol. Biol.* 703 107–122. 10.1007/978-1-59745-248-9_8 21125486

[B67] Zhang B. Liu Z. X. Ma J. Song Y. Chen F. J. (2015). Alternative splicing of the AGAMOUS orthologous gene in double flower of *Magnolia stellata* (Magnoliaceae). *Plant Sci.* 241 277–285. 10.1016/j.plantsci.2015.10.017 26706078

[B68] Zhang J. Kobert K. Flouri T. Stamatakis A. (2014). PEAR: a fast and accurate illumina paired-end reAd mergeR. *Bioinformatics* 30 614–620. 10.1093/bioinformatics/btt593 24142950 PMC3933873

