# Supplementary material for: Integrative Analysis of Three RNA Sequencing Methods Identifies Mutually Exclusive Exons of MADS-Box Isoforms During Early Bud Development in Picea abies
Source: Front Plant Sci. 2018 Nov 13;9:1625. doi: 10.3389/fpls.2018.01625 (PMC6243048; doi:10.3389/fpls.2018.01625)
Supplement: Supplementary file 2 [file Data_Sheet_1.pdf]

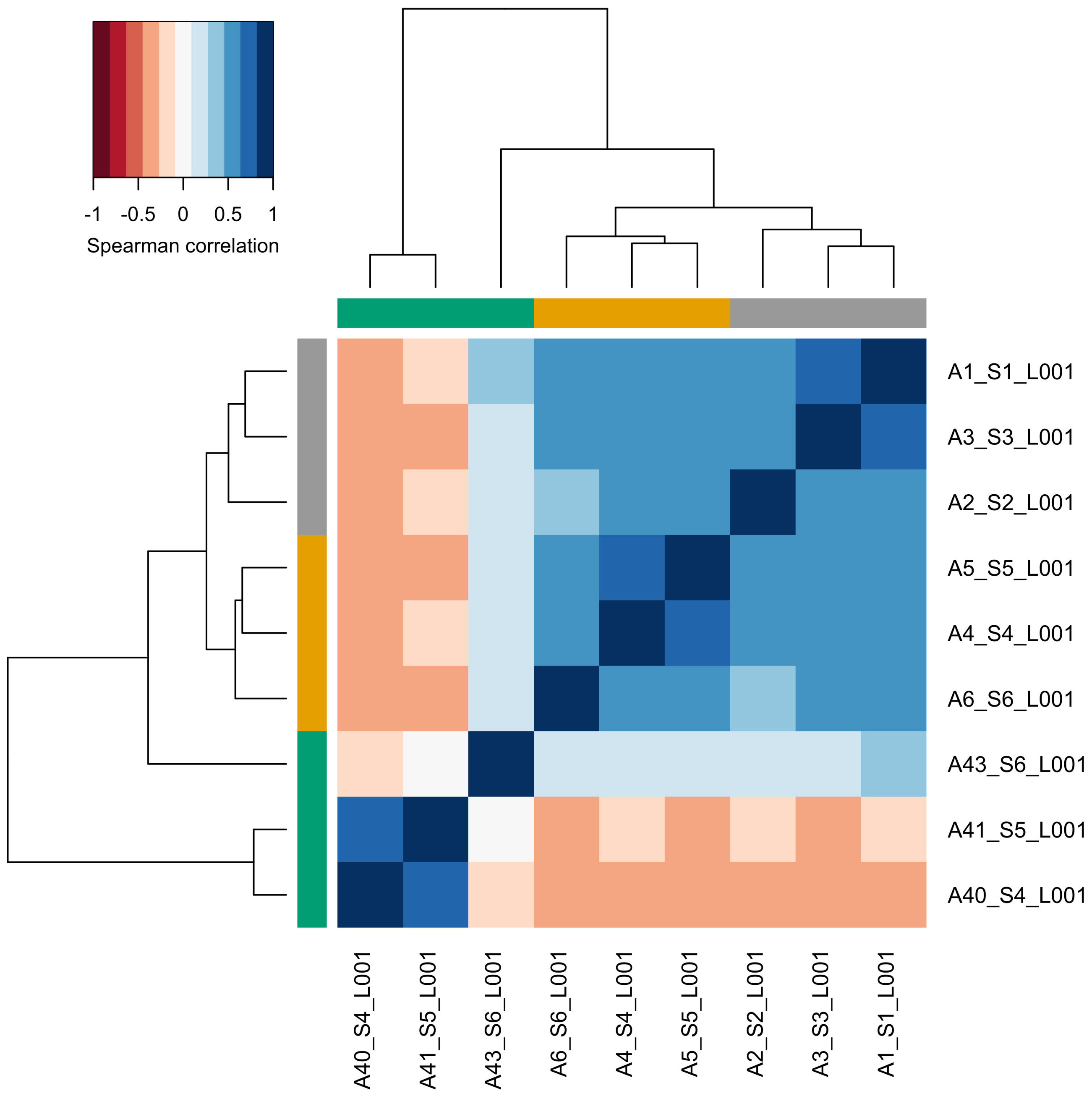

Supplemental Figure S1. Heatmap representation of Spearman correlation of nine bud samples based on the expression of 823 transcripts that have a maximum read count greater than one. Samples are grouped by hierarchical clustering on Euclidean distance using read counts. Colored bars below and to the right of the top and left dendrogram, respectively, indicate bud sample type: Green, orange, and grey represent the bud types male, vegetative, and female, respectively. The male sample "A43_S6_L001" has relatively low correlation with all other samples.

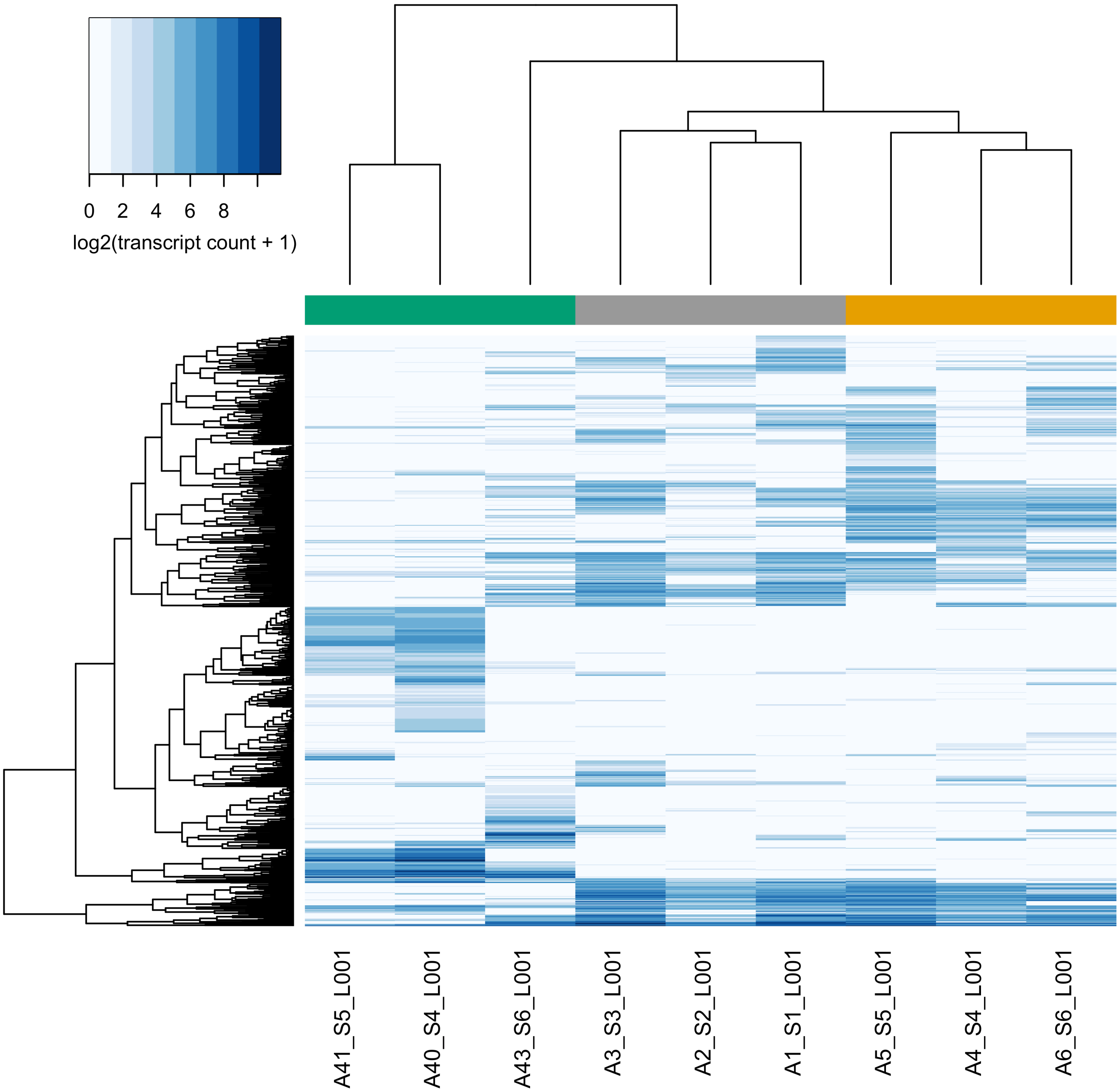

Supplemental Figure S2. Heatmap representation of log2(transcript count + 1) of 823 transcripts that have a maximum read count greater than one. Samples are grouped by hierarchical clustering of Euclidean distance. Colored bars below and to the right of the top and left dendrogram, respectively, indicate bud sample type: Green, orange, and grey represent the bud types male, vegetative, and female, respectively. The male sample "A43_S6_L001" has an expression pattern that does not match the pattern of any other sample.

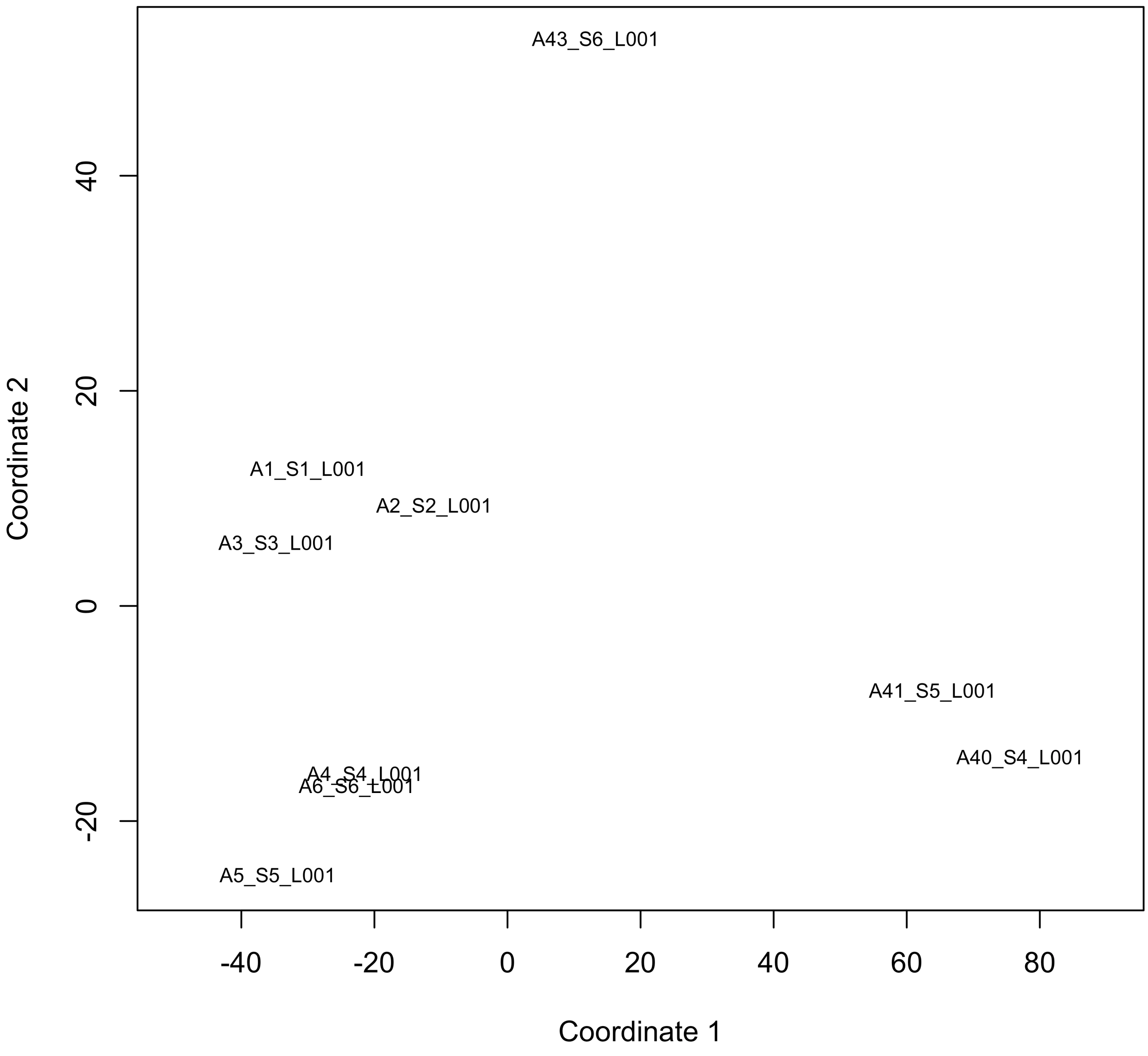

Supplemental Figure S3. Multi-dimensional scaling of nine bud samples using the Euclidean distance of the transformed expression of 823 transcripts that have a maximum read count greater than one. The transformation used is log2(transcript count + 1). Samples cluster into bud types, except for the male sample “A43_S6_L001”.

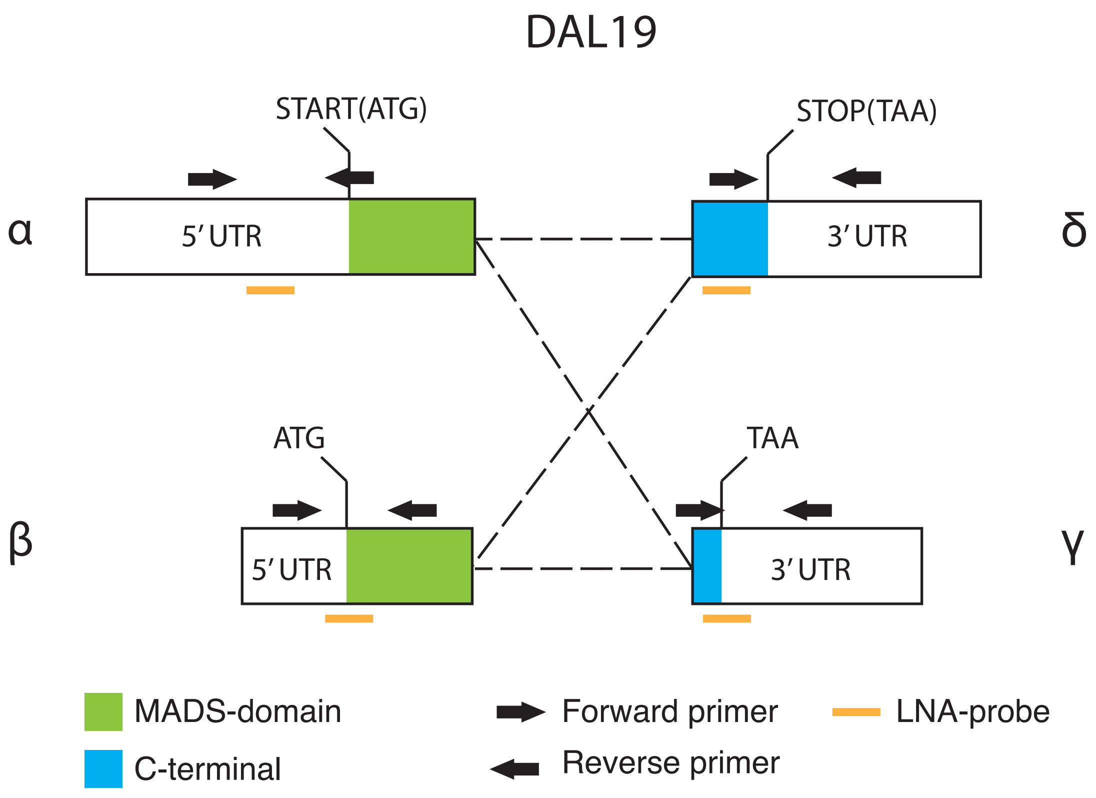

Supplemental Figure S4. Location of primers and probes: the position of forward and reverse primers (arrowheads) and LNA-probes (orange line) relative to the start codon in exons α and β, and relative to the stop codon in exons γ and δ. Note that assaying transcription of a *DAL19* exon does not provide direct evidence of which full-length isoform is transcribed. For example, *DAL19_ α* can either be combined with *DAL19_γ* or *DAL19_δ*. The core region Ψ is omitted in the figure and is only represented by interrupted lines.

Supplemental Figure S5

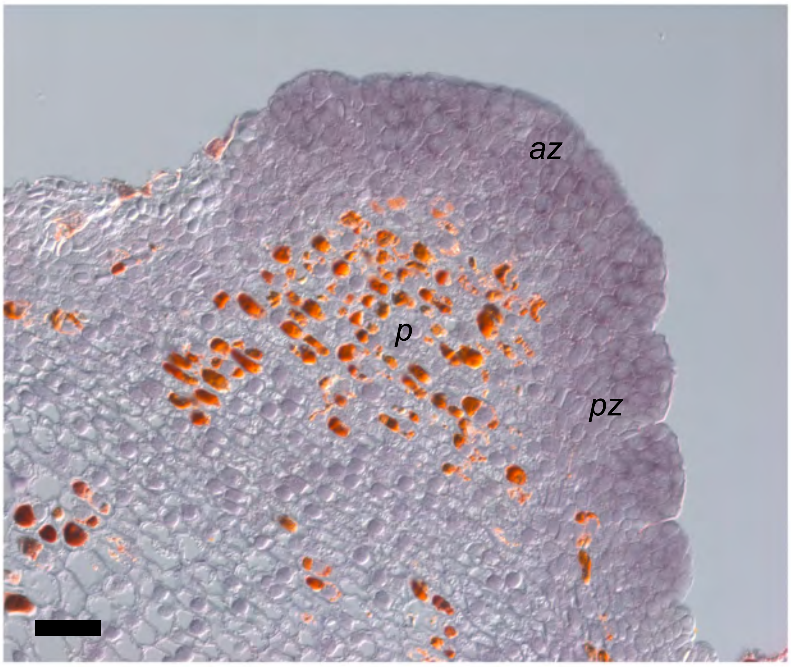

Supplemental Figure S5 mRNA in situ hybridization on longitudinal section of a vegetative bud meristem using a histon H2A specific LNA probe. Signals appear as purple patches in dividing cells.p=central pith, az= apical zone of the shoot apical meristem, pz= periferal zone of the meristem. Size bar = 50 *μ*m

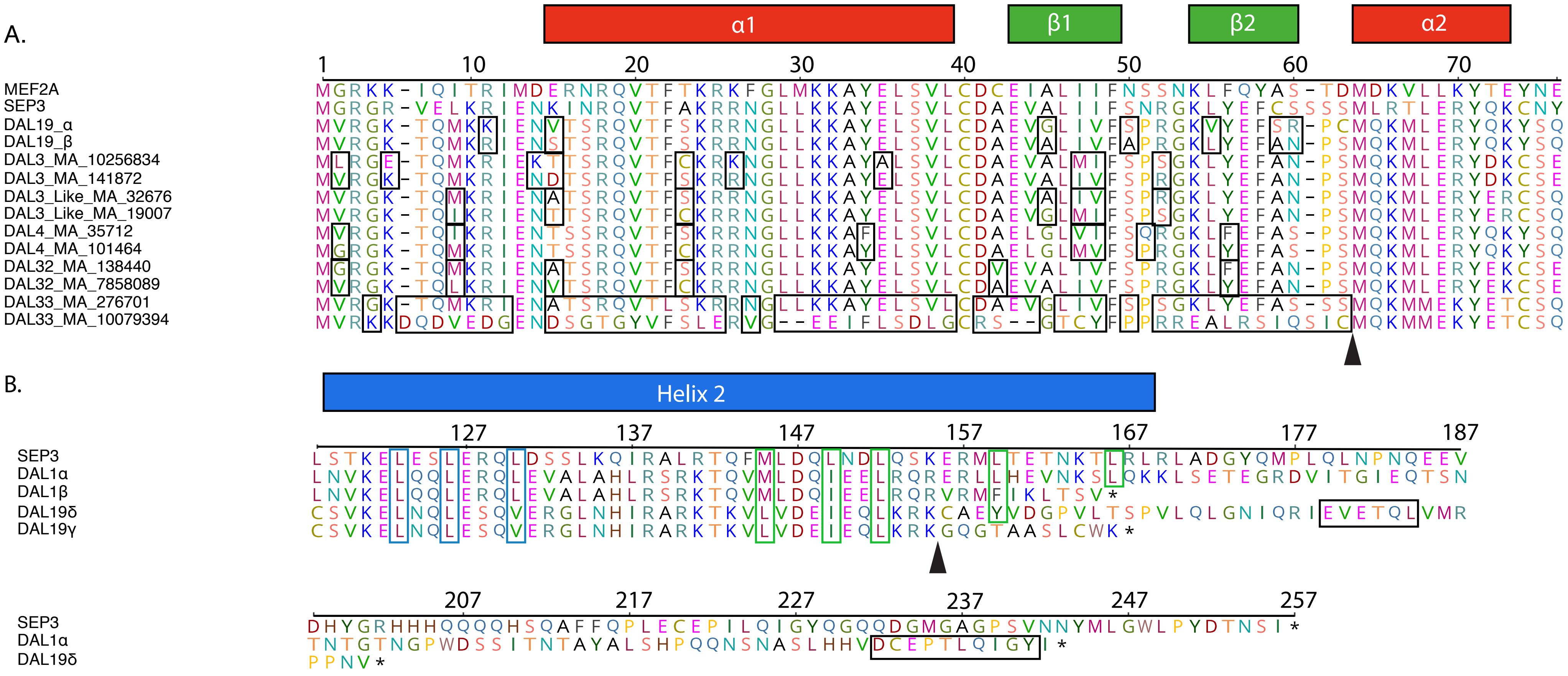

Supplemental Figure S6. Alignments of MADS-domains and C-terminal motifs. The figure shows the amino acid alignments of the MADS-domain in A) and the C-terminal motif in B). In A), isoforms of DAL-proteins are aligned to MEF2A from Homo sapiens which have been structurally characterized (Santelli and Richmond, 2000) and SEP3 from A. thaliana. Red and green boxes above the alignment indicate the of position alpha helixes and β- sheets in the MEF2A MADS-domain. In the alignment amino acids that differ between two isoforms are boxed. In C, 3′ isoforms of DAL1 and DAL19 are aligned to SEP3 for which the intervening region and the K-domain have been structurally characterized (Puranik et al., 2014). The blue box above the alignment indicates the position of the second helix in the SEP3 K-domain. Light blue boxes mark the position of hydrophobic amino acids of importance for dimerization. Green boxes mark the position of hydrophobic amino acids of importance for tetramerization. Black boxes mark the position of a TM3-signature motif in DAL19δ and an AGL6-motif in DAL1α. Arrowheads indicate the position of intron/exon borders.

References:

Puranik, S., Acajjaoui, S., Conn, S., Costa, L., Conn, V., Vial, A., Marcellin, R., Melzer, R., Brown, E., Hart, D., Theissen, G., Silva, C.S., Parcy, F., Dumas, R., Nanao, M., and Zubieta, C. (2014). Structural basis for the oligomerization of the MADS domain transcription factor SEPALLATA3 in Arabidopsis. Plant Cell 26, 3603-3615.
Santelli, E., and Richmond, T.J. (2000). Crystal structure of MEF2A core bound to DNA at 1.5 A resolution. J Mol Biol 297, 437-449.

Supplemental Figure S7 Phylograms from Phylogenetic analyses of the MADS-box gene family using the nucleotide sequence of complete Open Reading Frame (ORFs) in A) and the nucleotide sequence of only the MADS-domains in B). Heuristic searches of 1000 random stepwise additions resulted in one most parsimonious tree of length 27487 (tree scores CI= 0.167, RI=0.481, RC= 0.080, HI= 0.833) in A) and one most parsimonious tree of length 3548 (tree scores CI= 0.210, RI=0.608, RC= 0.128, HI= 0.789) in B). Red numbers indicate bootstrap support for nodes in the trees. Genes from *Arabidopsis thaliana* genes are color coded in blue, *Lycopersicon esculentum* in purple and gymnosperm gene names are given in black. Sub-clades named by founder genes are indicated. Note that genes belonging to the AP1-clade are sometimes also referred to as SQUA-like genes, and that genes belonging to the AP3/PI- clade sometimes are referred to as DEF/GLO-like genes, see *e.g.* Gramzow et.al. (2014). Accession numbers for the sequences included in the analysis are provided in Supplemental Table S4.

ORF

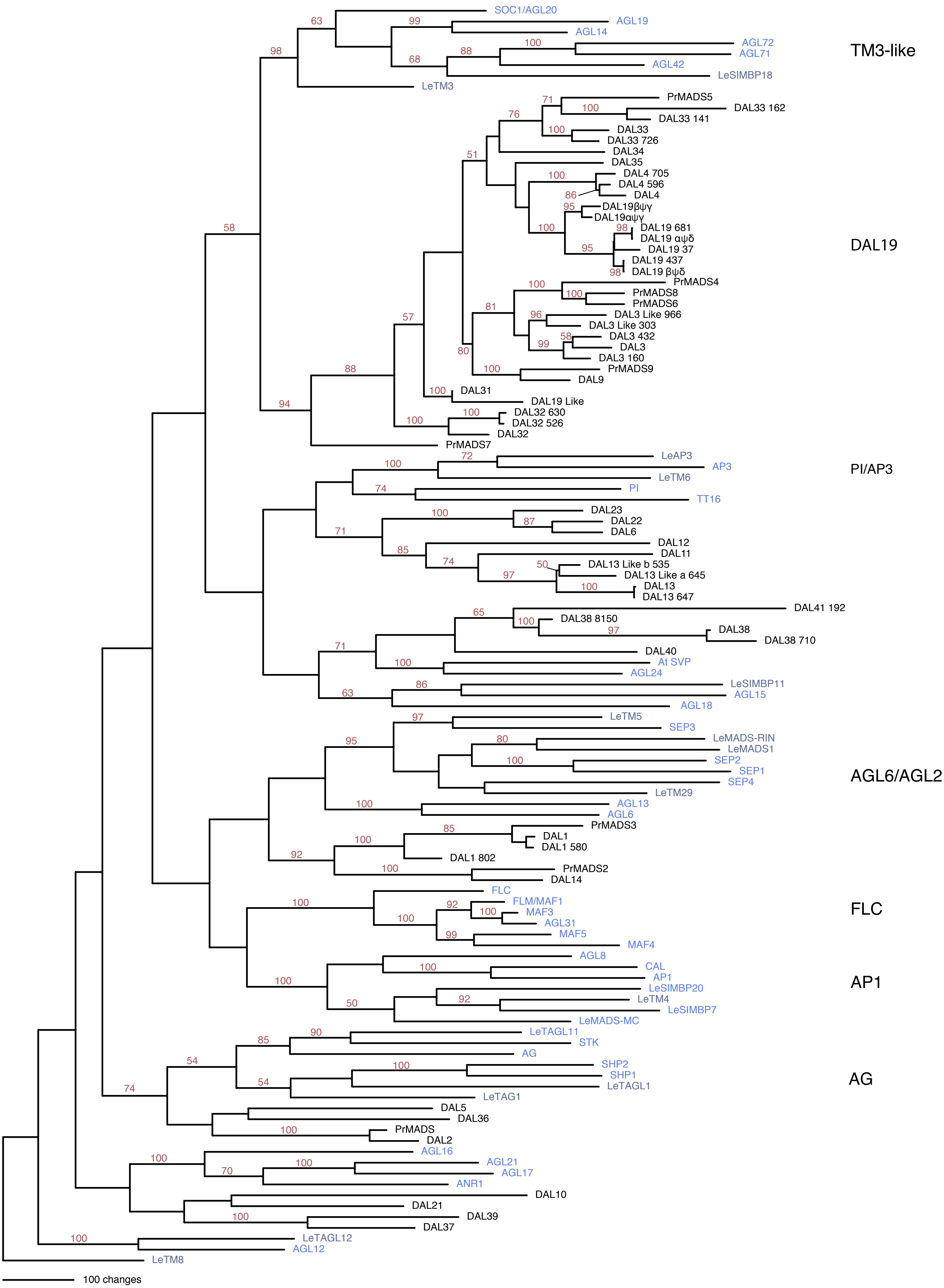

Supplemental Figure S7A

B. Only MADS-box

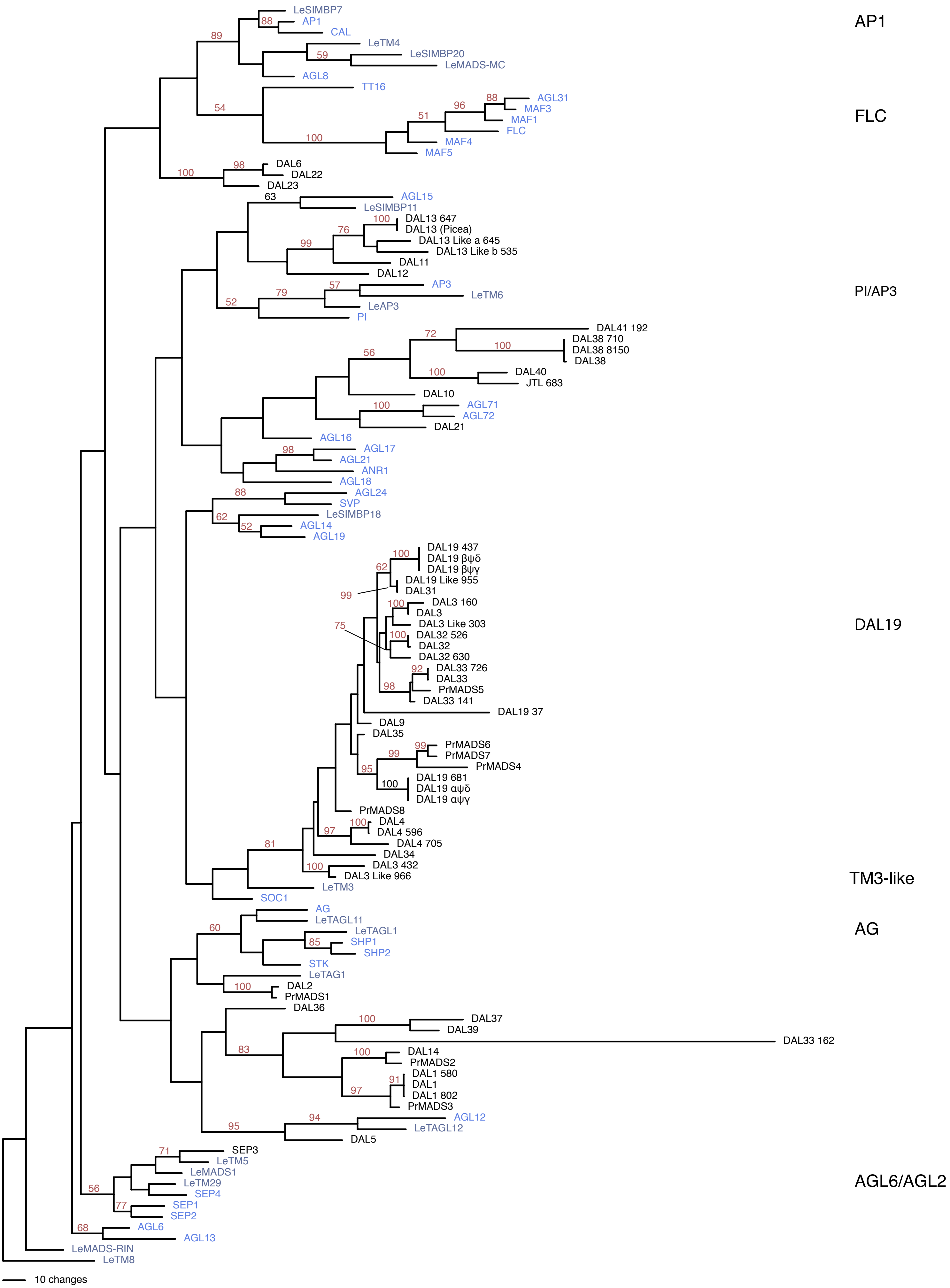

Supplemental Figure S7B
