# Supplementary material for: Integrative Analysis of Three RNA Sequencing Methods Identifies Mutually Exclusive Exons of MADS-Box Isoforms During Early Bud Development in Picea abies
Source: Front Plant Sci. 2018 Nov 13;9:1625. doi: 10.3389/fpls.2018.01625 (PMC6243048; doi:10.3389/fpls.2018.01625)
Supplement: Supplementary file 3 [file Data_Sheet_2.PDF]

Supplemental listings of commands

Removal of rRNA with SortMeRNA

```
sortmerna --ref \
sortmerna/rRNA_databases/rfam-5s-database-id98.fasta,\
sortmerna/index/rfam-5s-database-id98:\
sortmerna/rRNA_databases/rfam-5.8s-database-id98.fasta,\
sortmerna/index/rfam-5.8s-database-id98:\
sortmerna/rRNA_databases/silva-arc-16s-id95.fasta,\
sortmerna/index/silva-arc-16s-id95:\
sortmerna/rRNA_databases/silva-bac-16s-id90.fasta,\
sortmerna/index/silva-bac-16s-id90:\
sortmerna/rRNA_databases/silva-euk-18s-id95.fasta,\
sortmerna/index/silva-euk-18s-id95:\
sortmerna/rRNA_databases/silva-arc-23s-id98.fasta,\
sortmerna/index/silva-arc-23s-id98:\
sortmerna/rRNA_databases/silva-bac-23s-id98.fasta,\
sortmerna/index/silva-bac-23s-id98:\
sortmerna/rRNA_databases/silva-euk-28s-id98.fasta,\
sortmerna/index/silva-euk-28s-id98 \
--reads totRNA-18_S32_L004_1 --other totRNA-18_S32_L004_sortmerna.fastq \
fastq --paired_in --fastx -a 16 --sam --num_alignments 1 \
--aligned totRNA-18_S32_L004_sortmerna_rRNA
```

Trimming of RNA-seq reads with Trimmomatic

```
java -jar \
  trimmomatic_dir/trimmomatic.jar PE -threads 16 -phred33 \
  totRNA-18_S32_L004_sortmerna_1.fq.gz totRNA-18_S32_L004_sortmerna_2.fq.gz \
  out_1.fq out_1.unpaired.fq out_2.fq out_2.unpaired.fq \
  ILLUMINACLIP:trimmomatic_dir/adapters/TruSeq3-PE-2.fa:2:30:10 \
  SLIDINGWINDOW:5:20 MINLEN:50
```

Graph traversal with Cortexpy

```
cortexpy traverse \
  TATGCAAAAAATGTTGGAGAGGTATCAAAAGTATTCACAAGAAAGTG \
  --graphs input_graph.ctx \
  --max-nodes 20000 \
  --out - | \
```

cortexpy view traversal - --to-json > output.json

Assembly with Trinity

Trinity --full_cleanup --KMER_SIZE 25 --seqType fq \
 --left totRNA-18_S32_L004_sortmerna_1.fq.gz \
 --right totRNA-18_S32_L004_sortmerna_2.fq.gz \
 --output trinity_output_dir

Assembly with Oases log

Fri Feb 9 22:08:10 2018
 velveth /crex2/proj/sllstore2017083/nobackup/winni/proj/olof/spruce/results/2018-02-09_assembly/b2015052_nobackup/assembly/trimmomatic/oases 47 -shortPaired -fastq -separate /crex2/proj/sllstore2017083/nobackup/winni/proj/olof/spruce/results/2018-02-09_assembly/b2015052_nobackup/assembly/trimmomatic/pear/totRNA-18_S32_L004/pear.unassembled.forward.fq.gz /crex2/proj/sllstore2017083/nobackup/winni/proj/olof/spruce/results/2018-02-09_assembly/b2015052_nobackup/assembly/trimmomatic/pear/totRNA-18_S32_L004/pear.unassembled.reverse.fq.gz -short2 -fastq /crex2/proj/sllstore2017083/nobackup/winni/proj/olof/spruce/results/2018-02-09_assembly/b2015052_nobackup/assembly/trimmomatic/pear/totRNA-18_S32_L004/pear.assembled.fq.gz
Version 1.2.10
Copyright 2007, 2008 Daniel Zerbino (zerbino@ebi.ac.uk)
This is free software; see the source for copying conditions. There is NO
warranty; not even for MERCHANTABILITY or FITNESS FOR A PARTICULAR PURPOSE.
Compilation settings:
CATEGORIES = 2
MAXKMERLENGTH = 64
OPENMP

Fri Feb 9 22:19:35 2018
 velvetg /crex2/proj/sllstore2017083/nobackup/winni/proj/olof/spruce/results/2018-02-09_assembly/b2015052_nobackup/assembly/trimmomatic/oases -read_trkg yes
Version 1.2.10
Copyright 2007, 2008 Daniel Zerbino (zerbino@ebi.ac.uk)
This is free software; see the source for copying conditions. There is NO
warranty; not even for MERCHANTABILITY or FITNESS FOR A PARTICULAR PURPOSE.
Compilation settings:
CATEGORIES = 2
MAXKMERLENGTH = 64

OPENMP

Final graph has 364990 nodes and n50 of 248, max 5334, total 37418260, using 4654554/5941280 reads
Fri Feb 9 22:37:07 2018
 oases /crex2/proj/sllstore2017083/nobackup/winni/proj/olof/spruce/results/2018-02-09_assembly/b2015052_nobackup/assembly/trimmomatic/oases -ins_length2 500
Version 1.2.10
Copyright 2007, 2008 Daniel Zerbino (zerbino@ebi.ac.uk)
This is free software; see the source for copying conditions. There is NO
warranty; not even for MERCHANTABILITY or FITNESS FOR A PARTICULAR PURPOSE.
Compilation settings:
CATEGORIES = 2
MAXKMERLENGTH = 64

Finished extracting transcripts, used 5552009/5941280 reads on 22533 loci
